# Molecular signatures of differential responses to exercise trainings during rehabilitation

**DOI:** 10.15761/BGG.1000127

**Published:** 2017-04-10

**Authors:** Yi-Wen Chen, Chris Gregory, Fan Ye, Naoe Harafuji, Donovan Lott, San-Huei Lai, Sunita Mathur, Mark Scarborough, Parker Gibbs, Celine Baligand, Krista Vandenborne

**Affiliations:** 1 Research Center for Genetic Medicine, Children’s National Medical Center, Washington DC, USA; 2 Department of Integrative Systems Biology, George Washington University, Washington DC, USA; 3 Department of Health Sciences and Research, Medical University of South Carolina, Charleston, SC, USA; 4 Department of Applied Physiology and Kinesiology, University of Florida, Gainesville, FL, USA; 5 Department of Physical Therapy, University of Florida, Gainesville, FL, USA; 6 Department of Physical Therapy, University of Toronto, Toronto, Ontario, USA; 7 Department of Orthopaedics and Rehabilitation, University of Florida, Gainesville, FL, USA; 8 Department of Physiology and Functional Genomics, University of Florida, Gainesville, FL, USA

**Keywords:** resistance training, microarray, atrophy, mitochondria, heat shock proteins

## Abstract

The loss and recovery of muscle mass and function following injury and during rehabilitation varies among individuals. While recent expression profiling studies have illustrated transcriptomic responses to muscle disuse and remodeling, how these changes contribute to the physiological responses are not clear. In this study, we quantified the effects of immobilization and subsequent rehabilitation training on muscle size and identified molecular pathways associated with muscle responsiveness in an orthopaedic patient cohort study. The injured leg of 16 individuals with ankle injury was immobilized for a minimum of 4 weeks, followed by a 6-week rehabilitation program. The maximal cross-sectional area (CSA) of the medial gastrocnemius muscle of the immobilized and control legs were determined by T1-weighted axial MRI images. Genome-wide mRNA profiling data were used to identify molecular signatures that distinguish the patients who responded to immobilization and rehabilitation and those who were considered minimal responders. RESULTS: Using 6% change as the threshold to define responsiveness, a greater degree of changes in muscle size was noted in high responders (−14.9 ± 3.6%) compared to low responders (0.1 ± 0.0%) during immobilization. In addition, a greater degree of changes in muscle size was observed in high responders (20.5 ± 3.2%) compared to low responders (2.5 ± 0.9%) at 6-week rehabilitation. Microarray analysis showed a higher number of genes differentially expressed in the responders compared to low responders in general; with more expression changes observed at the acute stage of rehabilitation in both groups. Pathways analysis revealed top molecular pathways differentially affected in the groups, including genes involved in mitochondrial function, protein turn over, integrin signaling and inflammation. This study confirmed the extent of muscle atrophy due to immobilization and recovery by exercise training is associated with distinct remodeling signature, which can potentially be used for evaluating and predicting clinical outcomes.

## Introduction

Skeletal muscle remodeling occurs in response to physiological stimuli, affecting both muscle size and functional properties. Skeletal muscle atrophy occurs during muscle disuse, such as immobilization, mircrogravity as well as bed rest. The reported decrease in muscle size during 2 to 6 weeks of disuse ranges from 0 to 26% depending on the duration of disuse, the modality used and the muscles studied [1–4].

Many molecular processes have been reported to be involved in muscle atrophy, including pathways affecting inflammation, mitochondrial function, protein turn over, extracellular matrix remodeling and autophagy [5–10]. Although considerable attention has focused on muscle atrophy with disuse, less attention has centered on muscle recovery. A small number of studies performed in healthy volunteers subjected to either unloading or cast immobilization, reported that the recovery time following disuse is approximately equal to the duration of disuse. Less consensus exists on the rate of recovery in patient populations. Most of the studies show that recovery following an orthopaedic injury is often incomplete despite intense rehabilitation interventions [11–14]. In addition, considerable inter-individual variation in the response to rehabilitation treatment exists, leading investigators to group patients into “responders” and “non-responders” [15,16].

The development of effective therapeutic interventions necessitates an in depth understanding of molecular mechanisms mediating muscle atrophy and the subsequent recovery. Disuse causes significant muscle remodeling, including a loss of myofibrillar proteins, a shift in metabolic profile from slow to fast, and vascular and neural alterations [5,17,18]. The rapid loss in myofibrillar protein during disuse is mediated by a transient decrease in protein synthesis, followed by an increase in protein degradation, resulting in net protein loss [5,19–21]. Gene regulating protein turnover including heat shock proteins and genes involved in protein degradation have been reported as one of the major pathways involved in muscle atrophy [22–25].

There is strong evidence that the ubiquitin-proteasome proteolytic pathway is the primary system responsible for the breakdown of long-lived skeletal muscle proteins [8,26,27] and that the ubiquitin protein ligases E3 (e.g. atrogin-1 or FBX32) define much of its specificity [28–30]. In addition, genes involved in mitochondrial functions have been reported to be severely affected in different conditions leading to muscle atrophy including immobilization, unloading, space flight and cancer cachexia [5,23,31–35] although the direct trigger of the changes is not clear.

Conversely, increase in neuromuscular activity (e.g. during high resistance training) induces an increase in protein content, myonuclear number, myofiber size and muscle strength; created in part via a cycle of injury and muscle regeneration and remodeling. Muscle regeneration is a complex process, requiring the coordinated interaction between myogenic progenitor cells or satellite cells, growth factors, protein synthesis, cytokines, capillary morphogenesis, and the extracellular matrix [36]. Previous studies showed genes and molecular pathways involved in energy metabolism, protein ubiquitination, extracellular matrix remodeling, vasculature remodeling and inflammation are involved in muscle responses to resistant training [22,25,37–43].

Although considerable progress has been made towards defining the pathophysiology of muscle regeneration and hypertrophy, it should be pointed out that most of our knowledge today is based on information extrapolated from animal models and exercise training studies in healthy and aged individuals. Few studies have specifically investigated the pathophysiology of muscle remodeling and rehabilitation in patient populations. In this study, we hypothesize that the key molecular pathways involved in muscle remodeling during atrophy and rehabilitation are differentially activated in “responders” versus “non-responders”. In this study, we temporal expression profiled muscle biopsies obtained from two groups of orthopaedic patients that showed different responsiveness to cast immobilization and a standardized rehabilitation intervention. The expression profiles of the two groups were compared to identify molecular signatures that distinguish responders from non-responders.

## Methods

### Participants

Twenty-four individuals (13 men, 11 women; mean age 26.7 ± 8.3 years) participated in this study (Table 1). Of these 24 subjects, 16 participated in all of the tests and measures at all of the four time points (see next paragraph). These 16 were included in the gene profiling portion of the study. All subjects had sustained an injury to the lower leg that was treated conservatively (4–6 weeks of immobilization). Following immobilization, each subject completed 6 weeks of a structured rehabilitation program focused on progressive resistance training of the ankle plantarflexor musculature as previously described [14]. The study was approved by the institutional review board at the University of Florida and Children’s National Medical Center. All subjects provided their signed and informed consent before participating in the study.

### Muscle cross-sectional area

Magnetic resonance imaging (MRI) was performed on either a 1.5T (Signa, GE Medical Systems) or 3.0T (Achieva, Philips Medical Systems) whole-body scanner at the following time points: at the end of the 4–6 week immobilization period for a baseline measure, (pre-rehab), 3 weeks into the rehabilitation program (mid-rehab), following the 6 week rehabilitation program, (post-rehab), and 6 months after immobilization (control). Subjects were placed in a supine position with their lower leg positioned in either a lower-extremity quadrature coil (1.5T) or an 8-channel sensitivity encoding, receive-only extremity coil (3.0T). MRI was done on both lower legs for each subject covering the area from the tibial tuberosity to the proximal Achilles tendon. Three-dimensional transaxial fat-suppressed gradient echo images were acquired with an optimized field of view (12–16 cm^2^), matrix 231 × 231 pixels, and a 7mm slice thickness. Acquisition parameters were TR/TE=17.6/3.3 ms and flip angle=10° at 1.5T, and TR/TE=17.5/1.9 ms and flip angle=20° at 3.0T.

The head of the medial gastrocnemius was manually outlined using OsiriX Imaging Software, an open-source software. The axial image with the largest cross-sectional area (CSA) in the series was identified. The average of three consecutive slices (the slice with the greatest CSA, the slice below, and the slice above) was calculated to determine the maximal CSA (CSAmax) for the medial gastrocnemius. Based on the changes in CSAmax the subjects were grouped in high or low responders. Subjects with changes in CSAmax > 6% were categorized as a high response subject, <6% is defined as a low response subject. The cut-off value of 6% was selected by taking into account the reproducibility of the MR CSAmax measurements and the amount of atrophy that was considered clinically meaningful [14].

All statistical analyses were performed with SPSS for Windows, Version 16.0 (SPSS Inc., Chicago, IL). Independent t-tests were used to compare the maximal CSA between different groups. A significance level of p<0.05 was used for all comparisons. Data were presented as mean ± SEM.

### Muscle biopsy

A total of 4 biopsies were obtained at different time points (pre-rehab, mid-rehab, post-rehab and control) from the medial gastrocnemius. Skin was first sterilized and anesthetized with a 2% lidocaine hydrochloride solution. The orthopaedic surgeon then made a small incision (less than 1/4″) in the skin and fascia, a sterile biopsy needle was inserted, and a small amount of tissue was removed. All biopsy tissue was snap frozen in liquid nitrogen and stored at −80°C for further analysis.

### Expression profiling

Affymetrix Human Genome U133 Plus 2.0 microarrays containing approximately 47,000 transcripts were used for the expression profiling experiment. Standard procedures including total RNA isolation, cDNA synthesis, cRNA labeling, microarray hybridization and image acquisition were done as described in the manufacturer’s protocol and our previous publications [44,45]. Briefly, total RNA samples were isolated with TRIzol reagent (Invitrogen) then purified with RNeasy MinElute Cleanup Kit (Qiagen) following the manufacturer’s protocol. Two hundred nanograms of total RNA from each sample were reverse-transcribed to double-stranded cDNA followed by *in vitro* cRNA synthesis using one-cycle target labeling and control reagents and protocol (Affymetrix). Biotin-labeled cRNA was then purified using GeneChip^®^ Sample Cleanup Module (Affymetrix) and fragmented randomly prior to hybridizing to the microarrays over night. Each array was washed and stained using the Affymetrix Fluidics Station 450, and then scanned using the GeneChip^®^ Scanner 3000. The quality control criteria developed at Children’s National Medical Center Microarray Center for each array were followed [46].

The array image analysis was performed using Microarray Suite 5.0 (MAS 5.0) (Affymetrix, CA). After the absolute analysis, the gene expression values were imported into GeneSpring 11.0 (Silicon Genetics) for data filtering and statistical analysis. First, genes were filtered with numbers of present calls across the arrays analyzed. Genes with at least 7 present calls (detected by more than 10% of the arrays) were selected for statistical analysis. We identified 32,766 probe sets met this filtering criterion. In GeneSpring, paired t-test was performed and probe sets showing significant (p<0.05) expression changes were retained for pathway analysis.

To investigate molecular networks and pathways associated with gene lists in this study, Ingenuity Pathway Analysis (IPA) (Ingenuity Systems) was used to identify gene interactions and to prioritize molecular pathways differentially affected in different groups. The significance of the association between the genes in each dataset and the canonical pathway was determined by Fischer’s exact test. The p-values were calculated to determine the probability of the association between the genes and the pathways. All profiles are made publicly accessible via NCBI GEO (http://www.ncbi.nlm.nih.gov/geo/) #GSE45462. Hierarchical clustering was performed using GeneSpring software to visualize transcripts showing coordinated regulation as a function of time.

## Results

### Impact of immobilization and rehabilitation on maximum muscle CSA

**Figure 1:**
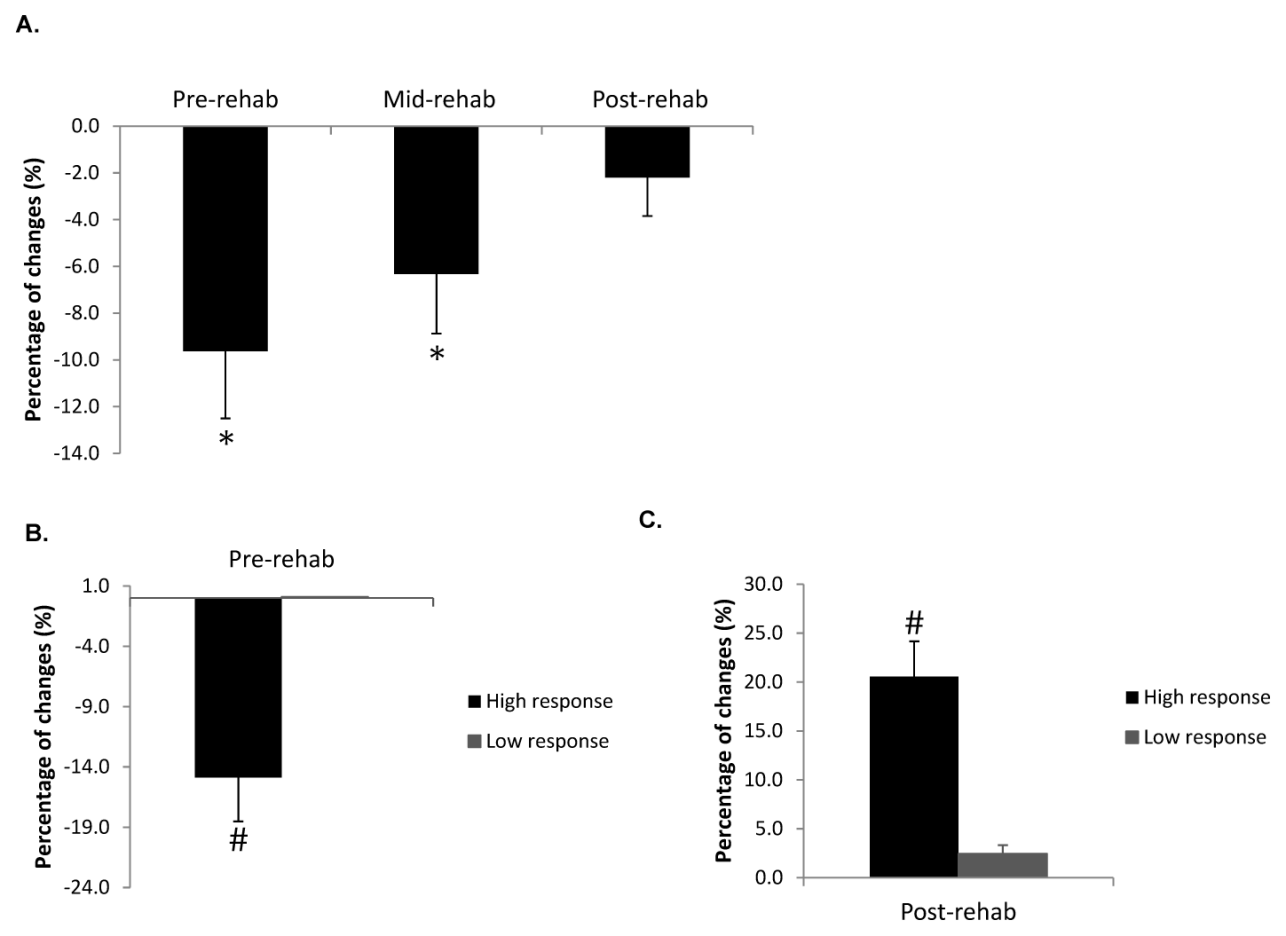
Percentage changes of maximal CSA (CSAmax) for the medial gastrocnemius from A) all the subjects at the first three time points; *Significantly different compared with control average CSA from the contralateral (uninvolved) legs. B) subjects included in the expression profiling study at pre-rehab in comparison with control average CSA from the contralateral (uninvolved) legs; # Significantly different compared with low responders (P<0.05). C) subjects included in the expression profiling study after 6-week rehabilitation program (post-rehab) in comparison with pre-rehab from the same (involved) sides. # Significantly different compared with low responders (P<0.05). Dataare presented as mean±SEM.

**Figure 2:**
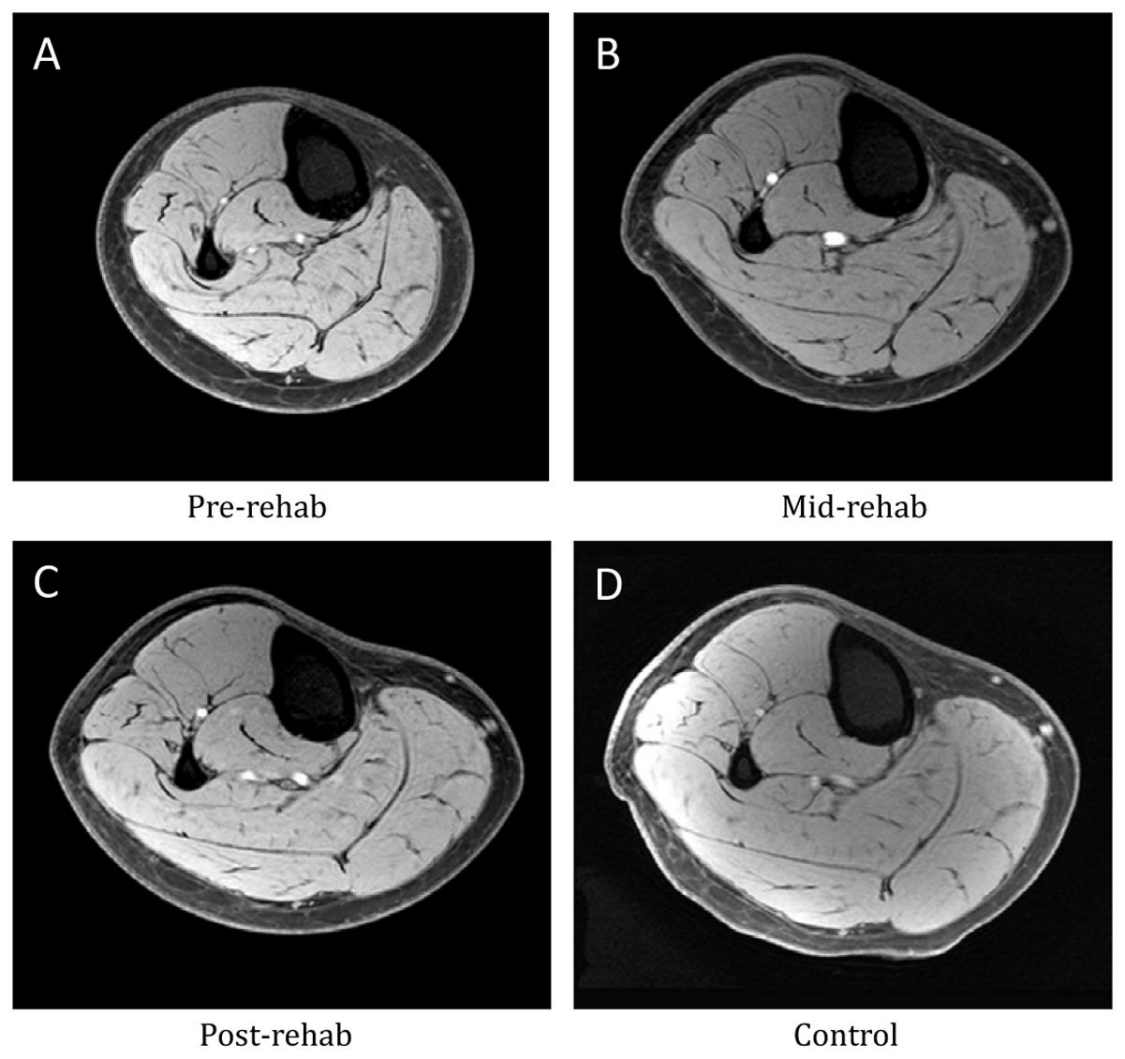
Representative transaxial proton T1-weighted MRIs of the lower leg from different time points of one patient in the high response group. (A) pre-rehab, (B) mid-rehab, (C) post-rehab and (D) control.

Overall, CSAmax of the medial gastrocnemius in the injured legs at pre-rehab was 9.6 ± 2.9% smaller compared to that of the un-injured legs. At mid-rehab the CSAmax had increased in size to being 6.3 ± 2.5% less than the un-injured legs (p<0.05). By the post-rehab time point (6 weeks), the CSAmax had returned to a level comparable to that of the un-injured leg (n=24, p>0.05, Figure 1A). We then further examined subjects that were included in the expression profiling study (n=16) and stratified them into high responders and low responders based on the extent of muscle atrophy during immobilization as well as the extent of muscle recovery during and after rehabilitation. High responders were defined as subjects who demonstrated more than 6% change in muscle size. Figures 1B and 1C illustrate the morphological changes for both groups separately, with low responders (n=4) showing relatively little atrophy (0.1 ± 0.0%) after the immobilization and high responders (n=8) showing a 14.9 ± 3.6% decrease in muscle size (Figure 1B, p<0.05). Conversely, in response to the rehabilitation intervention high responders showed a 20.5 ± 3.2% increase (n=9) in muscle size, while low responders showed little recovery (2.5 ± 0.9% increase, n=7) (Figure 1C, p<0.05). It is worth pointing out that, in general, low responders for changes in muscle size with immobilization were also low responders for rehabilitation, and high responders for immobilization were also high responders for rehabilitation. Figure 2 contains MRIs of the lower leg of one subject who responded to the immobilization and rehabilitation training, which demonstrate the change in muscle size over the four different time points.

### Global gene expression changes during immobilization and rehabilitation

To identify genes differentially expressed during immobilization (pre-rehab) and rehabilitation (mid- and post-rehab), we performed expression profiling using Affymetrix Human Genome U133 Plus 2.0 microarrays containing approximately 47,000 transcripts.

**Figure 3:**
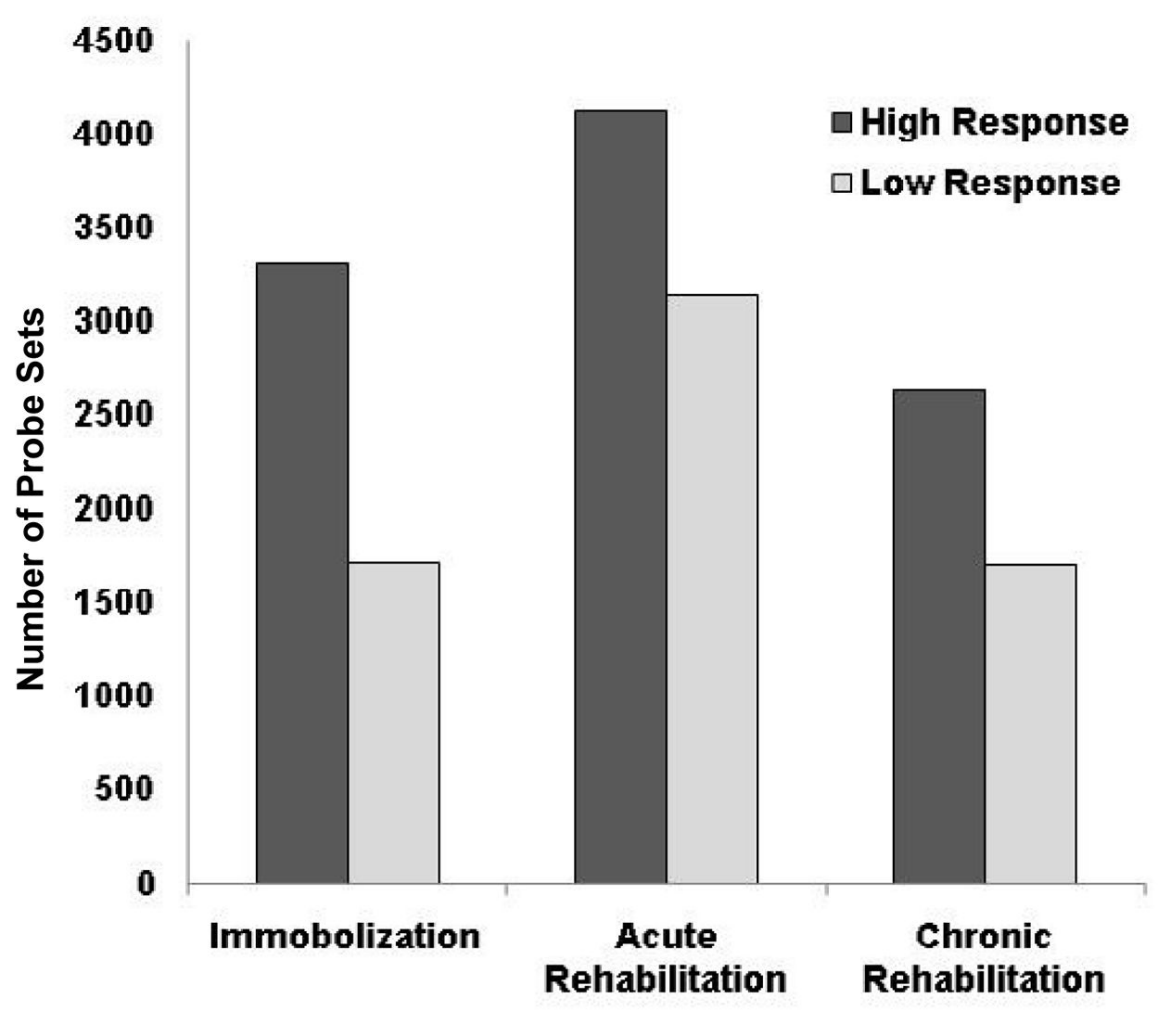
Patients in the high response group showed greater number of gene expression changes during immobilization (pre-rehab), early rehabilitation (3 weeks, mid-rehab) and late rehabilitation (6 weeks, post-rehab) stages. More expression changes during the acute stage of rehabilitation in both groups were identified in comparison to the other stages.

Filtered by Affymetrix “present calls”, transcripts corresponding to 32,766 probe sets were defined as detected by the arrays and retained for further analysis. Genes differentially regulated at each time point compared to the control time point of the same subjects were identified using paired t-tests. Note the control biopsy of each individual was collected 6 months after the completion of the rehabilitation to assess the baseline gene expression. In the high response group, 3313 probe sets were differentially expressed between the pre-rehab and the control time points, while changes of only 1710 probe sets were detected in the low response group (Figure 3). At the mid-rehab time point, 4124 and 3138 probe sets showed significant changes in the high and low response groups, respectively. At the post-rehab time point, 2627 and 1693 probe sets showed significant changes in the high and low response groups, respectively. The data showed an overall higher numbers of genes differentially expressed in the responders compared to non-responders at both the immobilization and the rehabilitation stages. Interestingly more expression changes were identified at the mid-rehab time points in both groups in comparison to the pre- and post-rehab time points.

### Distinct molecular signatures during immobilization and rehabilitation between the high and low response groups

To identify major molecular pathways affected in the two groups during the immobilization and rehabilitation, the probe sets showed significant changes were further analyzed using Ingenuity Pathway Analysis (IPA) and hierarchical clustering analysis.

Table 2 shows the top 5 ranked canonical pathways identified by IPA. Please note that while the names of the pathways often directly indicate their biological functions, tissue-specific roles of the changes were not taken into consideration when the pathways were constructed by the IPA, therefore a cancer related pathway may indicate a pathway involved in regulating cell growth and survival in skeletal muscles instead of cancer.

Subjects who responded to immobilization by reducing muscle mass significantly showed changes in pathways involving in mitochondrial function, protein turnover and BMP signaling (Table 2). Individual genes in each of the pathways are listed in supplemental table 1 (pre-rehab vs. control). The top ranked pathways in subjects who showed low responsiveness to the immobilization and rehabilitation were different from those of the high responders. The top 5 pathways include genes involving GM-CSF Signaling, Protein ubiquitination pathway, glioma signaling, FAK Signaling and CXCR4 Signaling (Table 2). Individual genes in each of the pathways are listed in supplemental table 2 (pre-rehab vs. control). While the only shared pathway between the two groups of subjects is the protein ubiquitination pathway, only a small number of genes in the pathway (8 transcripts) were shared between the two groups while others are different members involved in the pathway. In addition, while the ubiquitination pathway was highly ranked in the high response group during both mid- and post-rehabilitation, it was only highly ranked in the low response group following immobilization.

**Figure 4:**
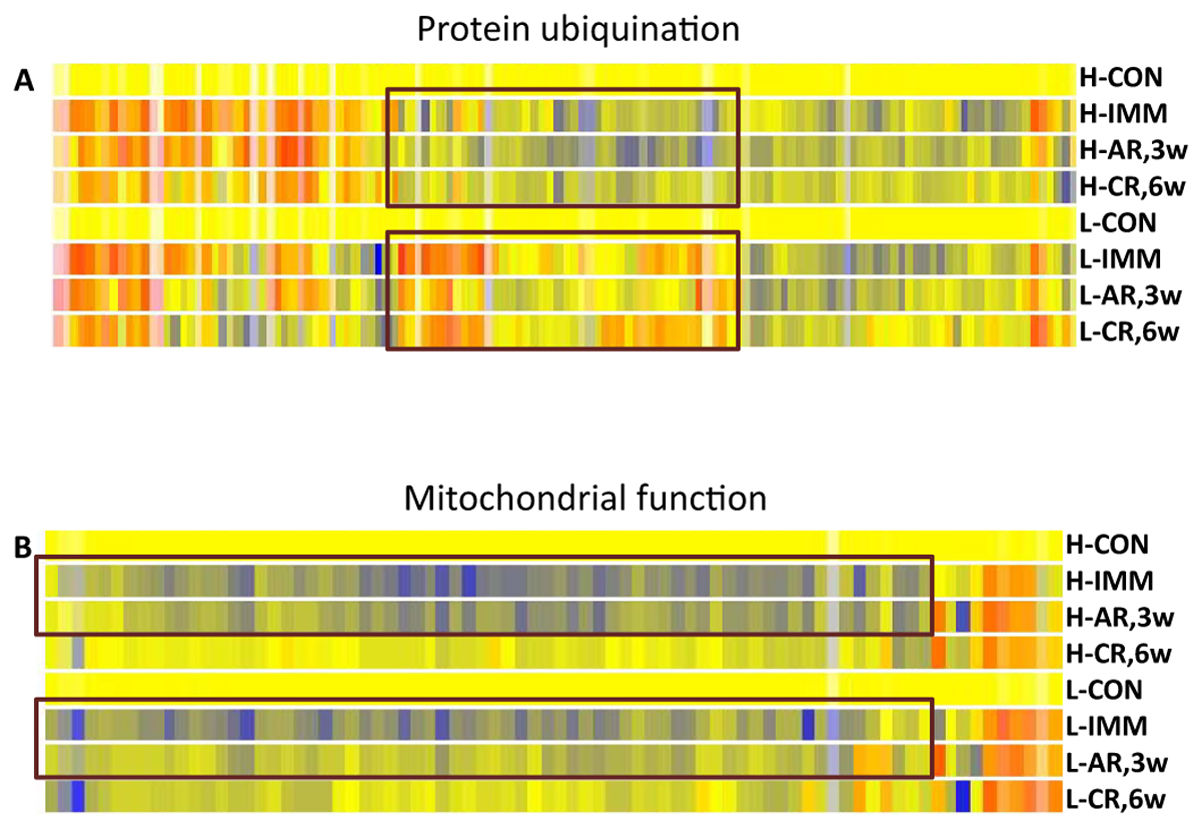
Distinct molecular signatures during immobilization and rehabilitation between subjects in the high and low response groups were identified by hierarchical clustering analysis During immobilization and rehabilitation periods, genes involved inmitochondrial functions were affected more in muscles of the high responders compared to those of low responders. B. A subset of stress response proteins was down-regulated in muscles of the high responders. Color code: yellow: baseline; red: up-regulated; blue: down-regulated. H:high responder; L:low responder; CON: control time point; IMM: pre- rehab/immobilization; AR, 3w: mid-rehab/3 weeks acute phase of rehabilitation; CR, 6w: post-rehab/6 weeks chronic phase of rehabilitation.

To visualize the expression pattern of the genes involved in ubiquitination pathway, we selected all the genes that were differentially expressed in either group at any of the 3 time points, followed by hierarchical clustering analysis. The analysis clustered genes with similar expression patterns at all time points therefore genes clustered together shared similar expression changes in each group. In contrast to the mitochondrial genes, figure 4A showed that many genes in the ubiquitination pathway were differentially expressed between the high and low responder groups. A cluster of genes that showed obvious opposite changes were circled (Figure 4A) and listed in supplemental table 3. One third (15/44) of the transcripts in the cluster belonged to heat shock protein family and one third (16/44) functioned in the ubiquitin proteasome pathways. The data showed a generally lower expression of these genes in the high responder group during the rehabilitation period although only the changes at the mid- rehab time point were significant (Table S3).

Among the pathways that are highly ranked during both mid- and post- rehabilitation, genes involved in integrin signaling were differentially expressed in both high and low response groups. While there are more genes in this category differentially expressed in the high responding group, there is no obvious difference in terms of expression pattern when visualized by hierarchical cluster analysis. The data suggested a stronger activation of the integrin signaling in the high responders while the pathway was activated in both group. This is supported by that the activation of the ILK signaling and the higher fold changes only in the high response group (Table S1 and S2).

Although many of the pathways were shared between the groups with either obvious or subtle differences, some pathways showed specific activation in one of the groups only. Pathways involved in antigen presentation pathway and Fcγ receptor-mediated phagocytosis in macrophages and monocytes were highly ranked at the acute stage of the rehabilitation in the high response group only, while actin cytoskeleton signaling pathway was activated in the low response group during the same stage of the rehabilitation (Table 1).

### Mitochondrial genes are highly regulated during immobilization and rehabilitation in muscles of the high responders

During immobilization the 3 top ranked pathways of the responders contain genes involved in mitochondrial functions. The majority of the genes in the pathways were down-regulated in muscles during immobilization. These genes include those involved in oxidative phosphorylation, ubiquinone biosynthesis and mitochondrial membrane proteins (Table 2 and Table S1). To visualize the expression patterns of these genes in the high and low responder groups, we performed hierarchical clustering analysis of the genes that were differentially expressed at any one of the 3 time points of both groups. Figure 4B showed that most of the genes were down-regulated in both groups during immobilization, however the changes in the low response groups was milder. In addition, the expression changes of the genes returned toward baseline faster in the low responders during the early rehabilitation stage (Figure 4B).

In the low responder group, genes involved in mitochondrial function were not highly ranked at the pre-rehab time point but some of the mitochondrial pathways moved up to the top ranked functional groups during the mid- and post-rehab time points. The data indicated that genes involved in mitochondrial functions were significantly down-regulated in the high responders while the mitochondrial changes were delayed in the low responder group and to a less extent in general. Instead, genes involved in AKT signaling pathways were activated in the muscles of low responders (Table S2).

## Discussion

Expression profiling has been used to examine transcriptomic changes in response to muscle immobilization and exercise training [22,24,25,39,47–50]. While individual studies of transcriptomic responses to conditions leading to muscle remodeling have been conducted, to our knowledge this is the first longitudinal study investigating both immobilization and rehabilitation stages in a patient population. Our data showed an overall higher numbers of genes differentially expressed in the responders compared to non-responders at both the immobilization and the rehabilitation stages, likely to directly reflect the activities of muscle remodeling. Interestingly more expression changes at the acute stage of rehabilitation in both groups were identified in comparison to those at the immobilization and chronic stages. When the differentially expressed genes were analyzed using IPA, distinct pathways were activated at each stage without major overlap between the responder and non-responder groups. The findings suggest that the remodeling is most active at the beginning of the rehabilitation. Instead of simply reversing the molecular changes due to muscle immobilization, additional pathways were activated to actively regain the lost muscles. In addition, the pathways involved in the whole process from the immobilization to rehabilitation are quite different between the high responder group and the low responder group, suggesting different molecular pathways were involved in the two groups of patients.

Based on genome-wide mRNA expression data, specific molecular signatures associated with high responsiveness and low responsiveness to immobilization and rehabilitation were identified. Not surprisingly, the expression of more transcripts was changed in the high responders, indicating that the responsiveness involved transcriptional regulation. Pathways analyses showed that distinct molecular pathways were affected in the two groups. During immobilization, the top-ranked molecular pathways affected in the high responders are involved in mitochondrial functions, protein turnover and BMP signaling. The down-regulation of mitochondrial genes has been reported previously to be a major change in muscle atrophy caused by different conditions including immobilization, unloading, space flight and cancer cachexia [5,23,31–35]. Genes regulating protein turnover including heat shock proteins and genes involved in protein degradation have also been reported as one of the major pathways involved in muscle atrophy [22–25]. Our findings suggest that the pathways and genes involved in mitochondrial function and protein degradation are critical responses involved in muscle atrophy. The BMP signaling pathway identified by IAP was not reported as a major molecular signature of responses to immobilization previously, although it is involved in limb development and muscle regeneration [51]. In this study, the two most up-regulated genes were follistatin (2.5 fold) and noggin (2.1 fold). Follistatin is a known positive regulator of muscle mass. It can inhibit myostatin activity by protein-protein interaction and promote muscle growth *in vivo* [52]. Noggin is an inhibitor of BMP signaling which has been shown critical to suppress premature differentiation of satellite cells [53]. Up-regulation of the noggin can potentially suppress the BMP signaling and promote regeneration. The findings suggest that these changes are likely to be compensatory responses as part of the effort to maintain the muscle mass, which might be critical for rapid recovery in the high responders when the rehabilitation starts.

The only shared pathway in both high and low responder groups was the protein ubiquitination pathway. Interestingly, while the pathway was affected significantly in both groups, only 8 genes were actually shared between the groups. When all the genes that were differentially expressed in either group were grouped together and analyzed by hierarchical clustering analysis, we showed that a subgroup of the genes consisted of heat shock proteins and genes involved in protein degradations was differentially regulated. These genes were down-regulated in the high responders but not changed in the low responders. Both the heat shock proteins and the genes involved in protein degradation have been shown critical to muscle regeneration and remodeling. Several heat shock proteins including HSP70 and HSP90 has been shown to be up-regulated in muscles in response to exercise and can improve muscle repair [54–59]. One of the HSP40 proteins, DNAJB6, was discovered as the causative gene of the limb-girdle muscular dystrophy type 1E [60]. While the role of HSP40 and HSP105 in muscle atrophy is not clear, several studies showed that these heat shock proteins can suppress the aggregation of truncated proteins with expanded CAG repeats and cell toxicity in neurodegenerative diseases likely by increasing in target protein degradation via the ubiquitin-proteasome system [61–63]. While the cause of the lower expression level of the heat shock proteins in the high responder group is not clear, this is an interesting molecular signature for the high responders and likely contributes to their susceptibility to more severe muscle atrophy during immobilization.

In the low response group, the 4 activated pathways that were not shared with the high response group were all centered at AKT/PI3K signaling which plays critical role in regulating cell proliferation, survival and metabolism. The PI3K/AKT/mTOR signaling pathway is critically involved in muscle survival and remodeling. Its role in muscle atrophy and hypertrophy in response to various stimuli has been extensively studied.

The activation of AKT signaling and its downstream targets glycogen synthase kinase- 3beta (GSK3beta), mTOR and Foxo1 have been shown to promote muscle hypertrophy and attenuate muscle atrophy [25,28,64,65]. While the AKT1 is the most studied form, the role of AKT2 and 3 in the muscles is less known. The biological significance of these signatures in the low responders needs to be further investigated.

Our study showed that the high responders and low responders have distinct molecular signatures during the rehabilitation process. During the acute phase of the rehabilitation the most highly ranked pathways in the high responders are pathways known to be involved in muscle responses to resistance training, including genes in the integrin and ILK signaling pathways as well as inflammation. Integrin and ILK signaling pathways have been shown to play critical roles in both cardiac and skeletal muscle hypertrophy [22,37,38,40,41,43]. In our study, 6 integrins were up-regulated in the high responder group with most of them previously reported to be involved in angiogenesis or muscle differentiation [66–72]. One of the integrins, integrin alpha 4 (2.3 fold), was reported to mediate BMP activated ILK signaling. Integrin-linked kinase (ILK) is a serine/threonine kinase and scaffolding protein [73]. ILK has been implicated in cancer cell growth and survival by modulating AKT signaling.

Skeletal muscle expresses high levels of ILK at myotendinous junctions and costameres, which stabilizes myotendinous junctions and protects muscle from stress-induced damage [74]. Previous studies also showed that ILK functions as a molecular adaptor protein linking integrins to the actin cytoskeleton and regulating actin polymerization [75–77]. Mice lacking ILK develop phenotypes resembling those seen in humans and mice lacking the alpha 7-integrin subunit, which lead to muscular dystrophy [78]. In addition to the roles of integrin and ILK signaling in skeletal muscle cells, some of the integrins identified are known to play a critical role in angiogenesis, which is a major process affecting skeletal muscle remodeling during muscle repair and hypertrophy [76,79,80]. In addition, these pathways and the protein ubiquitination pathways have been shown to be up-regulated in muscles in response to exercise. Our findings showed that the activation of integrin and ILK pathways, indicative of active muscle remodeling and angiogenesis, is a molecular signature in the high responder group only. An attenuated response was observed in the low responder group, suggesting a weaker muscle remodeling response.

Inflammation including cellular infiltration and specific cytokine productions has been shown to play key roles in muscle hypertrophy in response to acute exercise [39,81–83]. In addition, it is known that inflammation plays a critical role in muscle regeneration [84,85]. In our study, we identified genes and pathways involved in inflammation in the high responder group only at the mid-rehab stage. The inflammatory responses were not detected during the later phase of rehabilitation as expected. Therefore, the activation of inflammatory pathways at the beginning of rehabilitation appeared to be unique molecular signature of the high responder group.

While genes involved in integrin signaling were also ranked in the top 5 of the low responders during rehabilitation, the other four pathways created a distinct signature of the molecular responses of the low responders during the acute phase of training. Instead of inflammatory and extracellular matrix remodeling, the signature indicated a continuation of mitochondrial dysfunction in these subjects. This mitochondrial defect remained at the top of all pathways affected even at the later stage of rehabilitation suggesting the defect in mitochondrial function and energy production may play a major role in the low responsiveness in these subjects. In addition, the results of the study showed that changes of these molecular pathways can potentially be used as molecular markers to determine the effects of rehabilitation and evaluate the patients’ responsiveness to the training.

Longitudinal studies of muscle dysfunction with disuse in healthy subjects indicate the extent of muscle atrophy is muscle specific. In the present study, 4 weeks of cast immobilization induced a decrease in plantarflexor muscle size, which is consistent with the literature [2,86]. Interestingly, the adults in this study showed a range of variability in their responsiveness to immobilization and training. Factors not under investigation in the present study have been demonstrated to affect the degree of muscle adaptations, such as age, sex, diet, activities of daily living, motivation, and previous training status [87,88]. Even when the above factors are controlled, the results of clinical studies still demonstrated considerable variation in the extent of muscle adaptations [89,90]. In addition, the compliance of the subjects with the rehabilitation program may also be a factor in how skeletal muscle tissue responds [91]. It may be useful to record the factors determining the adherence to the rehabilitation programs to assess or explain the differential responses/outcomes. Therefore, more information is needed to help define what factors substantially influence the inter-subject variation in muscular adaption.

## Conclusion

Expression analysis based on the patients’ responsiveness to immobilization and rehabilitation yielded two major findings. First, training outcomes were positively correlated with the extent of transcriptomic changes. Individuals showed less adaptation to exercise also showed less gene expression changes in the muscle biopsies. Second, the molecular signatures of individuals who showed different responsiveness to immobilization and rehabilitation were distinct such as pathways involved in mitochondrial functions, protein turn over and muscle remodeling. These results suggest that these transcriptomic signatures of muscle gene expression may have predictive value in training outcomes in adults receiving rehabilitation following atrophy of skeletal muscle from disuse.

## Supplementary Material

Table S1

Table S2

Table S3

## Figures and Tables

**Table 1:** Characteristics of subjects included in gene analysis.

Subject Code	Gender	Age (years)	Height (cm)	Weight (kg)	BMI	Injury Type
**1**	F	20	170.2	86.2	29.8	fibular fracture
**2**	M	23	175.3	72.6	23.7	fibular fracture
**3**	M	28	177.8	93.9	29.8	metatarsal fracture
**4**	M	25	172.7	68.0	22.9	ankle sprain/avulsion of ATF on tibia
**5**	M	34	182.9	95.3	28.5	metatarsal fracture
**6**	M	44	172.7	104.3	35.0	fibular fracture
**7**	M	40	182.9	99.8	29.9	fibular fracture
**8**	M	22	175.3	65.3	21.3	metatarsal fracture
**9**	M	24	180.3	77.1	23.8	Unknown
**10**	M	29	180.3	81.6	25.2	metatarsal fracture
**11**	M	21	177.8	68.0	21.6	fibular fracture
**12**	M	46	190.5	83.9	23.2	calcaneal fracture
**13**	F	25	157.5	47.6	19.2	stress fracture
**14**	F	40	167.6	65.8	23.5	fibular fracture
**15**	F	20	170.2	68.0	23.5	distal fibular fracture
**16**	F	22	157.5	54.4	22.0	fibular fracture

**Table 2:** Top 5 canonical pathways affected in subjects during immobilization and rehabilitation. Number of significant probes in each pathway is included in the parentheses

Immobilization	High Responders	Low Responders
	Oxidative Phosphorylation (66)	GM-CSF Signaling (15)
	Mitochondrial Dysfunction (55)	Protein Ubiquitination Pathway (40)
	Ubiquinone Biosynthesis (33)	Glioma Signaling (18)
	Protein Ubiquitination Pathway (64)	FAK Signaling (17)
	BMP signaling pathway (25)	CXCR4 Signaling (24)
**Rehabilitation**	**High Responders**	**Low Responders**
	Protein Ubiquitination Pathway (84)	Mitochondrial Dysfunction (45)
	Fcγ Receptor-mediated Phagocytosis in Macrophages and Monocytes (31)	Oxidative Phosphorylation (44)
**Acute phase**		
	ILK Signaling (50)	Actin Cytoskeleton Signaling (55)
	Integrin Signaling (51)	Integrin Signaling (49)
	Antigen Presentation Pathway (15)	Ubiquinone Biosynthesis (22)
**Rehabilitation**	**High Responders**	**Low Responders**
**Chronic Phase**	Molecular Mechanisms of Cancer (58)	Mitochondrial Dysfunction (23)
	ILK Signaling (35)	Integrin Signaling (30)
	Clathrin-mediated Endocytosis Signaling (31)	Ephrin Receptor Signaling (24)
	Integrin Signaling (35)	HMGB1 Signaling (16)
	Protein Ubiquitination Pathway (43)	Rac Signaling (17)

## References

[1] Berg HE, Larsson L, Tesch PA (1997). Lower limb skeletal muscle function after 6 wk of bed rest. J Appl Physiol (1985).

[2] LeBlanc A, Gogia P, Schneider V, Krebs J, Schonfeld E (1988). Calf muscle area and strength changes after five weeks of horizontal bed rest. Am J Sports Med.

[3] Stevens JE, Pathare NC, Tillman SM, Scarborough MT, Gibbs CP (2006). Relative contributions of muscle activation and muscle size to plantarflexor torque during rehabilitation after immobilization. J Orthop Res.

[4] Tesch PA, Trieschmann JT, Ekberg A (2004). Hypertrophy of chronically unloaded muscle subjected to resistance exercise. J Appl Physiol (1985).

[5] Abadi A, Glover EI, Isfort RJ, Raha S, Safdar A (2009). Limb immobilization induces a coordinate down-regulation of mitochondrial and other metabolic pathways in men and women. PLoS One.

[6] Bialek P, Morris C, Parkington J, Andre MS, Owens J (2011). Distinct protein degradation profiles are induced by different disuse models of skeletal muscle atrophy. Physiol Genomics.

[7] Kemp GJ, Crowe AV, Anijeet HKI, Gong QY, Bimson WE (2004). Abnormal mitochondrial function and muscle wasting, but normal contractile efficiency, haemodialysed patients studied non-invasively *in vivo*. Nephrol Dial Transplant.

[8] Lecker SH, Jagoe RT, Gilbert A, Gomes M, Baracos V (2004). Multiple types of skeletal muscle atrophy involve a common program of changes in gene expression. FASEB J.

[9] Mammucari C, Milan G, Romanello V, Masiero E, Rudolf R (2007). FoxO3 controls autophagy in skeletal muscle *in vivo*. Cell Metab.

[10] Murton A, Constantin D, Greenhaff P (2008). The involvement of the ubiquitin proteasome system in human skeletal muscle remodelling and atrophy. Biochim Biophys Acta.

[11] Gaston P, Will EM, Keating JF (2005). Recovery of knee function following fracture of the tibial plateau. J Bone Joint Surg Br.

[12] Ingemann-Hansen T, Halkjaer-Kristensen J (1985). Physical training of the hypotrophic quadriceps muscle in a man, I: the effects of different training programs on muscular performance. Scand J Rehabil Med Suppl.

[13] Lewek M, Rudolph K, Axe M, Snyder-Mackler L (2002). The effect of insufficient quadriceps strength on gait after anterior cruciate ligament reconstruction. Clin Biomech (Bristol, Avon).

[14] Stevens JE, Walter GA, Okereke E, Scarborough MT, Esterhai JL (2004). Muscle adaptations with immobilization and rehabilitation after ankle fracture. Med Sci Sports Exerc.

[15] Gorassini MA, Norton JA, Nevett-Duchcherer J, Roy FD, Yang JF (2009). Changes in locomotor muscle activity after treadmill training in subjects with incomplete spinal cord injury. J Neurophysiol.

[16] Troosters T, Gosselink R, Decramer M (2001). Exercise training in COPD: how to distinguish responders from nonresponders. J Cardiopulm Rehabil Prev.

[17] Clark BC, Issac LC, Lane JL, Damron LA, Hoffman RL (2008). Neuromuscular plasticity during and following 3 wk of human forearm cast immobilization. J Appl Physiol (1985).

[18] Liu M, Stevens-Lapsley JE, Jayaraman A, Ye F, Conover C (2010). Impact of treadmill locomotor training on skeletal muscle IGF1 and myogenic regulatory factors in spinal cord injured rats. Eur J Appl Physiol.

[19] Booth F, Criswell D (1997). Molecular events underlying skeletal muscle atrophy and the development of effective countermeasures. Int J Sports Med.

[20] de Boer MD, Selby A, Atherton P, Smith K, Seynnes OR (2007). The temporal responses of protein synthesis, gene expression and cell signalling in human quadriceps muscle and patellar tendon to disuse. J Physiol.

[21] Ikemoto M, Nikawa T, Takeda S, Watanabe C, Kitano T (2001). Space shuttle flight (STS-90) enhances degradation of rat myosin heavy chain in association activation of ubiquitin–proteasome pathway. FASEB J.

[22] Chen YW, Gregory CM, Scarborough MT, Shi R, Walter GA (2007). Transcriptional pathways associated with skeletal muscle disuse atrophy in humans. Physiol Genomics.

[23] Nikawa T, Ishidoh K, Hirasaka K, Ishihara I, Ikemoto M (2004). Skeletal muscle gene expression in space-flown rats. FASEB J.

[24] Reich KA, Chen YW, Thompson PD, Hoffman EP, Clarkson PM (2010). 48 hours of Unloading and 24 hours of Reloading Leads to Changes in Global Gene Expression Patterns Related to Ubiquitination and Oxidative Stress in Humans. J Appl Physiol.

[25] Urso ML, Scrimgeour AG, Chen YW, Thompson PD, Clarkson PM (2006). Analysis of human skeletal muscle after 48 h immobilization reveals alterations in mRNA and protein for extracellular matrix components. J Appl Physiol.

[26] Attaix D, Ventadour S, Codran A, Bechet D, Taillandier D (2005). The ubiquitin-proteasome system and skeletal muscle wasting. Essays Biochem.

[27] Glickman MH, Ciechanover A (2002). The ubiquitin-proteasome proteolytic pathway: destruction for the sake of construction. Physiol Reviews.

[28] Bodine SC, Latres E, Baumhueter S, Lai VK, Nunez L (2001). Identification of ubiquitin ligases required for skeletal muscle atrophy. Science.

[29] Foletta VC, White LJ, Larsen AE, Léger B, Russell AP (2011). The role and regulation of MAFbx/atrogin-1 and MuRF1 in skeletal muscle atrophy. Pflugers Archiv.

[30] Gomes MD, Lecker SH, Jagoe RT, Navon A, Goldberg AL (2001). Atrogin-1, a muscle-specific F-box protein highly expressed during muscle atrophy. Proceedings of the National Academy of Sciences.

[31] Bera S, Ray M (2009). The transcriptional cascade associated with creatine kinase down-regulation and mitochondrial biogenesis in mice sarcoma. Cell Mol Biol Lett.

[32] Kim JW, Kwon OY, Kim MH (2007). Differentially expressed genes and morphological changes during lengthened immobilization in rat soleus muscle. Differentiation.

[33] Ohira Y, Yasui W, Kariya F, Wakatsuki T, Nakamura K (1994). Metabolic adaptation of skeletal muscles to gravitational unloading. Acta Astronaut.

[34] Oishi Y, Ogata T, Yamamoto KI, Terada M, Ohira T (2008). Cellular adaptations in soleus muscle during recovery after hindlimb unloading. Acta Physiol (Oxf).

[35] Shenkman BS, Nemirovskaya TL, Belozerova IN, Mazin MG, Matveeva OA (2002). Mitochondrial adaptations in skeletal muscle cells in mammals exposed to gravitational unloading. J Gravit Physiol.

[36] Goetsch SC, Hawke TJ, Gallardo TD, Richardson JA, Garry DJ (2003). Transcriptional profiling and regulation of the extracellular matrix during muscle regeneration. Physiological genomics.

[37] Boppart MD, Volker SE, Alexander N, Burkin DJ, Kaufman SJ (2008). Exercise promotes alpha7 integrin gene transcription and protection of skeletal muscle. Am J Physiol Regul Integr Comp Physiol.

[38] Brancaccio M, Hirsch E, Notte A, Selvetella G, Lembo G (2006). Integrin signalling: the tug-of-war in heart hypertrophy. Cardiovasc Res.

[39] Chen YW, Hubal MJ, Hoffman EP, Thompson PD, Clarkson PM (2003). Molecular responses of human muscle to eccentric exercise. J Appl Physiol (1985).

[40] Dallabrida SM, Ismail NS, Pravda EA, Parodi EM, Dickie R (2008). Integrin binding angiopoietin-1 monomers reduce cardiac hypertrophy. FASEB J.

[41] Johnston RK, Balasubramanian S, Kasiganesan H, Baicu CF, Zile MR (2009). Beta3 integrin-mediated ubiquitination activates survival signaling during myocardial hypertrophy. FASEB J.

[42] Kostek MC, Chen YW, Cuthbertson DJ, Shi R, Fedele MJ (2007). Gene expression responses over 24 h to lengthening and shortening contractions in human muscle: major changes in CSRP3, MUSTN1, SIX1, and FBXO32. Physiol Genomics.

[43] Lueders TN, Zou K, Huntsman HD, Meador B, Mahmassani Z (2011). The α7β1-integrin accelerates fiber hypertrophy and myogenesis following a single bout of eccentric exercise. Am J Physiol Cell Physiol.

[44] Chen YW, Zhao P, Borup R, Hoffman EP (2000). Expression profiling in the muscular dystrophies: identification of novel aspects of molecular pathophysiology. J Cell Biol.

[45] Dixit M, Ansseau E, Tassin A, Winokur S, Shi R (2007). DUX4, a candidate gene of facioscapulohumeral muscular dystrophy, encodes a transcriptional activator of PITX1. Proc Natl Acad Sci U S A.

[46] Tumor Analysis Best Practices Working Group (2004). Expression profiling--best practices for data generation and interpretation in clinical trials. Nat Rev Genet.

[47] Bye A, Høydal MA, Catalucci D, Langaas M, Kemi OJ (2008). Gene expression profiling of skeletal muscle in exercise-trained and sedentary rats with inborn high and low VO2max. Physiol Genomics.

[48] Mahoney DJ, Safdar A, Parise G, Melov S, Fu M (2008). Gene expression profiling in human skeletal muscle during recovery from eccentric exercise. Am J Physiol Regul Integr Comp Physiol.

[49] Pattison JS, Folk LC, Madsen RW, Childs TE, Spangenburg EE (2003). Expression profiling identifies dysregulation of myosin heavy chains IIb and IIx during limb immobilization in the soleus muscles of old rats. J Physiol.

[50] Urso ML, Chen YW, Scrimgeour AG, Lee PC, Lee KF (2007). Alterations in mRNA expression and protein products following spinal cord injury in humans. J Physiol.

[51] Clever JL, Sakai Y, Wang RA, Schneider DB (2010). Inefficient in inhibitor of differentiation knockout mice suggests a crucial role for BMP signaling during adult muscle regeneration. Am J Physiol Cell Physiol.

[52] Gilson H, Schakman O, Kalista S, Lause P, Tsuchida K (2009). Follistatin induces muscle hypertrophy through satellite cell proliferation and inhibition of both myostatin and activin. Am J Physiol Endocrinol Metab.

[53] Ono Y, Calhabeu F, Morgan JE, Katagiri T, Amthor H (2011). BMP signalling permits population expansion by preventing premature myogenic differentiation in muscle satellite cells. Cell Death Differ.

[54] Bornman L, Polla BS, Gericke GS (1996). Heat-shock protein 90 and ubiquitin: developmental regulation during myogenesis. Muscle Nerve.

[55] Chen YW, Nader GA, Baar KR, Fedele MJ, Hoffman EP (2002). Response of rat muscle to acute resistance exercise defined profiling. J Physiol.

[56] Gehrig SM, van der Poel C, Sayer TA, Schertzer JD, Henstridge DC (2012). Hsp72 preserves muscle function and slows progression of severe muscular dystrophy. Nature.

[57] Melling CW, Thorp DB, Milne KJ, Krause MP, Noble EG (2007). Exercise-mediated regulation of Hsp70 expression following aerobic exercise training. Am J Physiol Heart Circ Physiol.

[58] Paepe BD, Creus KK, Weis J, Bleecker JL (2012). Heat shock protein families 70 and 90 in Duchenne muscular dystrophy and inflammatory myopathy: balancing muscle protection and destruction. Neuromuscul Disord.

[59] Paulsen G, Hanssen KE, Ronnestad BR, Kvamme NH, Ugelstad I (2012). Strength training elevates HSP27, HSP70 and alphaB- crystallin levels in musculi vastus lateralis and trapezius. Eur J Appl Physiol.

[60] Sarparanta J, Jonson PH, Golzio C, Sandell S, Luque H (2012). Mutations affecting the cytoplasmic functions of the co- chaperone DNAJB6 cause limb-girdle muscular dystrophy. Nat Genet.

[61] Howarth JL, Kelly S, Keasey MP, Glover CP, Lee YB (2007). Hsp40 molecules that target to the ubiquitin-proteasome system decrease inclusion formation in models of polyglutamine disease. Mol Ther.

[62] Ishihara K, Yamagishi N, Saito Y, Adachi H, Kobayashi Y (2003). Hsp105alpha suppresses the aggregation of truncated androgen receptor with expanded CAG repeats and cell toxicity. J Biol Chem.

[63] Wyttenbach A, Carmichael J, Swartz J, Furlong RA, Narain Y (2000). Effects of heat shock, heat shock protein 40 (HDJ-2), and proteasome inhibition on protein aggregation in cellular models of Huntington’s disease. Proc Natl Acad Sci U S A.

[64] Lai KM, Gonzalez M, Poueymirou WT, Kline WO, Na E (2004). Conditional activation of akt in adult skeletal muscle induces rapid hypertrophy. Mol Cell Biol.

[65] Léger B, Cartoni R, Praz M, Lamon S, Dériaz O (2006). Akt signalling through GSK-3beta, mTOR and Foxo1 is involved in human skeletal muscle hypertrophy and atrophy. J Physiol.

[66] Lakhe-Reddy S, Khan S, Konieczkowski M, Jarad G, Wu KL (2006). Beta8 integrin binds Rho GDP dissociation inhibitor-1 and activates Rac1 to inhibit mesangial cell myofibroblast differentiation. J Biol Chem.

[67] Lehnert K, Ni J, Leung E, Gough S, Morris CM (1999). The integrin alpha10 subunit: expression pattern, partial gene structure, and chromosomal localization. Cytogenet Cell Genet.

[68] Leifheit-Nestler M, Conrad G, Heida NM, Limbourg A, Limbourg FP (2010). Overexpression of integrin beta 5 enhances the paracrine properties of circulating angiogenic cells via Src kinase-mediated activation of STAT3. Arterioscler Thromb Vasc Biol.

[69] Schwander M, Shirasaki R, Pfaff SL, Muller U (2004). Beta1 integrins in muscle, but not in motor neurons, are required for skeletal muscle innervation. J Neurosci.

[70] Tomczak KK, Marinescu VD, Ramoni MF, Sanoudou D, Montanaro F (2004). Expression profiling and identification of novel genes involved in myogenic differentiation. FASEB J.

[71] Wicik Z, Sadkowski T, Jank M, Motyl T (2011). The transcriptomic signature of myostatin inhibitory influence on the differentiation of mouse C2C12 myoblasts. Pol J Vet Sci.

[72] Yee KO, Rooney MM, Giachelli CM, Lord ST, Schwartz SM (1998). Role of beta1 and beta3 integrins in human smooth muscle cell adhesion to and contraction of fibrin clots *in vitro*. Circ Res.

[73] Perez VA, Ali Z, Alastalo TP, Ikeno F, Sawada H (2011). BMP promotes motility and represses growth of smooth muscle cells by activation of tandem Wnt pathways. J Cell Biol.

[74] Wang HV, Chang LW, Brixius K, Wickstrom SA, Montanez E, Thievessen I, Schwander M, Muller U, Bloch W, Mayer U, Fassler R (2008). Integrin-linked kinase stabilizes myotendinous junctions and protects muscle from stress-induced damage. J Cell Biol.

[75] Esfandiarei M, Yazdi SA, Gray V, Dedhar S, van Breemen C (2010). Integrin-linked kinase functions as a downstream signal of platelet-derived growth factor to regulate actin polymerization and vascular smooth muscle cell migration. BMC Cell Biol.

[76] Fan Y, Gong Y, Ghosh PK, Graham LM, Fox PL (2009). Spatial coordination of actin polymerization and ILK-Akt2 activity during endothelial cell migration. Dev Cell.

[77] Sakai T, Li S, Docheva D, Grashoff C, Sakai K (2003). Integrin-linked kinase (ILK) is required for polarizing the epiblast, cell adhesion, and controlling actin accumulation. Genes Dev.

[78] Gilson H, Schakman O, Kalista S, Lause P, Tsuchida K (2009). Follistatin induces muscle hypertrophy through satellite cell proliferation and inhibition of both myostatin and activin. Am J Physiol Endocrinol Metab.

[79] Tan C, Cruet-Hennequart S, Troussard A, Fazli L, Costello P (2004). Regulation of tumor angiogenesis by integrin-linked kinase (ILK). Cancer Cell.

[80] Xie J, Lu W, Gu R, Dai Q, Zong B (2011). The impairment of ILK related angiogenesis involved in cardiac maladaptation after infarction. PLoS One.

[81] MacIntyre DL, Sorichter S, Mair J, Berg A, McKenzie DC (2001). Markers of inflammation and myofibrillar proteins following eccentric exercise in humans. Eur J Appl Physiol.

[82] Nosaka K, Clarkson PM (1996). Changes in indicators of inflammation after eccentric exercise of the elbow flexors. Med Sci Sports Exerc.

[83] Sorichter S, Koller A, Haid C, Wicke K, Judmaier W (1995). Light concentric exercise and heavy eccentric muscle loading: effects on CK, MRI and markers of inflammation. Int J Sports Med.

[84] Contreras-Shannon V, Ochoa O, Reyes-Reyna SM, Sun D, Michalek JE (2007). Fat accumulation with altered inflammation and regeneration in skeletal muscle of CCR2−/− mice following ischemic injury. Am J Physiol Cell Physiol.

[85] Shireman PK, Contreras-Shannon V, Ochoa O, Karia BP, Michalek JE (2007). MCP-1 deficiency causes altered inflammation with impaired skeletal muscle regeneration. J Leukoc Biol.

[86] Akima H, Kuno S, Suzuki Y, Gunji A, Fukunaga T (1997). Effects of 20 days of bed rest on physiological cross-sectional area of human thigh and leg muscles evaluated by magnetic resonance imaging. J Gravitational Physiol.

[87] Folland JP, Williams AG (2007). The adaptations to strength training: morphological and neurological contributions to increased strength. Sports Med.

[88] Wernbom M, Augustsson J, Thomeé R (2007). The influence of frequency, intensity, volume and mode of strength training on whole muscle cross-sectional area in humans. Sports Med.

[89] Davidsen PK, Gallagher IJ, Hartman JW, Tarnopolsky MA, Dela F (2011). High responders to resistance exercise training demonstrate differential regulation of skeletal muscle microRNA expression. J Appl Physiol.

[90] Boppart MD, Volker SE, Alexander N, Burkin DJ, Kaufman SJ (2008). Exercise promotes alpha7 integrin gene transcription and protection of skeletal muscle. Am J Physiol Regul Integr Comp Physiol.

[91] Young P, Dewse M, Fergusson W, Kolbe J (1999). Respiratory rehabilitation in chronic obstructive pulmonary disease: predictors of nonadherence. Eur Respir J.

