# Supplementary material for: Molecular signatures of differential responses to exercise trainings during rehabilitation
Source: Biomed Genet Genom. Author manuscript; Available in PMC 2017 Aug 23. (PMC5568829; doi:10.15761/BGG.1000127)
Supplement: Table S1 [file NIHMS888767-supplement-Table_S1.pdf]

**Table S1**

**pre-rehab vs. control**

| Gene | Affymetrix accession # | Gene description |
|---|---|---|
| | Oxidative Phosphorylation | |
| ATP6V1A | 201971_s_at | ATPase, H+ transporting, lysosomal 70kDa, V1 subunit A |
| ATP6V0C | 36994_at | ATPase, H+ transporting, lysosomal 16kDa, V0 subunit c |
| SDHA | 201093_x_at | succinate dehydrogenase complex, subunit A, flavoprotein (Fp) |
| MT-CO2 | 1553570_x_at | mitochondrially encoded cytochrome c oxidase II |
| ATP6V0E1 | 200096_s_at | ATPase, H+ transporting, lysosomal 9kDa, V0 subunit e1 |
| PPA1 | 217848_s_at | pyrophosphatase (inorganic) 1 |
| MT-COI | 1553538_s_at | cytochrome c oxidase subunit I, Mitochondrion [Homo sapiens] |
| NDUFC1 | 203478_at | NADH dehydrogenase (ubiquinone) 1, subcomplex unknown, 1, 6kDa |
| NDUFA11 | 225304_s_at | NADH dehydrogenase (ubiquinone) 1 alpha subcomplex, 11, 14.7kDa |
| ATP5G2 | 208764_s_at | ATP synthase, H+ transporting, mitochondrial Fo complex, subunit C2 (subunit 9) |
| ATP5J2 | 202961_s_at | ATP5J2-PTCD1 readthrough |
| NDUFB7 | 202839_s_at | NADH dehydrogenase (ubiquinone) 1 beta subcomplex, 7, 18kDa |
| ATP5A1 | 213738_s_at | ATP synthase, H+ transporting, mitochondrial F1 complex, alpha subunit 1, cardiac muscle |
| NDUFS2 | 201966_at | NADH dehydrogenase (ubiquinone) Fe-S protein 2, 49kDa (NADH-coenzyme Q reductase) |
| NDUFB9 | 222992_s_at | NADH dehydrogenase (ubiquinone) 1 beta subcomplex, 9, 22kDa |
| ATP6V1D | 208898_at | ATPase, H+ transporting, lysosomal 34kDa, V1 subunit D |
| COX7B | 202110_at | cytochrome c oxidase subunit VIIb |
| NDUFC2 | 218101_s_at | NADH dehydrogenase (ubiquinone) 1, subcomplex unknown, 2, 14.5kDa |
| UQCRB | 205849_s_at | ubiquinol-cytochrome c reductase binding protein |
| NDUFS4 | 209303_at | NADH dehydrogenase (ubiquinone) Fe-S protein 4, 18kDa (NADH-coenzyme Q reductase) |
| NDUFA12 | 223244_s_at | NADH dehydrogenase (ubiquinone) 1 alpha subcomplex, 12 |
| ATP5B | 201322_at | ATP synthase, H+ transporting, mitochondrial F1 complex, beta polypeptide |
| ATP5F1 | 211755_s_at | ATP synthase, H+ transporting, mitochondrial Fo complex, subunit B1 |
| UQCRFS1 | 208909_at | ubiquinol-cytochrome c reductase, Rieske iron-sulfur polypeptide 1 |
| NDUFS6 | 203606_at | NADH dehydrogenase (ubiquinone) Fe-S protein 6, 13kDa (NADH-coenzyme Q reductase) |
| COX4I1 | 200086_s_at | cytochrome c oxidase subunit IV isoform 1 |
| NDUFB4 | 218226_s_at | NADH dehydrogenase (ubiquinone) 1 beta subcomplex, 4, 15kDa |
| NDUFB8 | 201226_at | NADH dehydrogenase (ubiquinone) 1 beta subcomplex, 8, 19kDa |
| NDUFB6 | 203613_s_at | NADH dehydrogenase (ubiquinone) 1 beta subcomplex, 6, 17kDa |
| NDUFA2 | 209224_s_at | NADH dehydrogenase (ubiquinone) 1 alpha subcomplex, 2, 8kDa |
| COX7C | 201134_x_at | cytochrome c oxidase subunit VIIc |
| NDUFB1 | 206790_s_at | NADH dehydrogenase (ubiquinone) 1 beta subcomplex, 1, 7kDa |
| UQCRQ | 201568_at | ubiquinol-cytochrome c reductase, complex III subunit VII, 9.5kDa |
| NDUFA13 | 220864_s_at | NADH dehydrogenase (ubiquinone) 1 alpha subcomplex, 13 |
| UQCRC2 | 200883_at | ubiquinol-cytochrome c reductase core protein II |

| | | |
|---|---|---|
| NDUFB5 | 203621_at | NADH dehydrogenase (ubiquinone) 1 beta subcomplex, 5, 16kDa |
| NDUFAB1 | 202077_at | NADH dehydrogenase (ubiquinone) 1, alpha/beta subcomplex, 1, 8kDa |
| NDUFA9 | 208969_at | NADH dehydrogenase (ubiquinone) 1 alpha subcomplex, 9, 39kDa |
| ATP5H | 210149_s_at | ATP synthase, H+ transporting, mitochondrial Fo complex, subunit d |
| NDUFB2 | 218200_s_at | NADH dehydrogenase (ubiquinone) 1 beta subcomplex, 2, 8kDa |
| ATP5O | 200818_at | ATP synthase, H+ transporting, mitochondrial F1 complex, O subunit |
| COX5A | 203663_s_at | cytochrome c oxidase subunit Va |
| NDUFA1 | 202298_at | NADH dehydrogenase (ubiquinone) 1 alpha subcomplex, 1, 7.5kDa |
| NDUFS8 | 203189_s_at | NADH dehydrogenase (ubiquinone) Fe-S protein 8, 23kDa (NADH-coenzyme Q reductase) |
| NDUFA8 | 218160_at | NADH dehydrogenase (ubiquinone) 1 alpha subcomplex, 8, 19kDa |
| NDUFS7 | 211752_s_at | NADH dehydrogenase (ubiquinone) Fe-S protein 7, 20kDa (NADH-coenzyme Q reductase) |
| SDHB | 202675_at | succinate dehydrogenase complex, subunit B, iron sulfur (Ip) |
| COX8A | 201119_s_at | cytochrome c oxidase subunit VIIIA (ubiquitous) |
| COX6B1 | 201441_at | cytochrome c oxidase subunit VIb polypeptide 1 (ubiquitous) |
| NDUFB11 | 218320_s_at | NADH dehydrogenase (ubiquinone) 1 beta subcomplex, 11, 17.3kDa |
| NDUFV1 | 208714_at | NADH dehydrogenase (ubiquinone) flavoprotein 1, 51kDa |
| ATP5L | 207573_x_at | ATP synthase, H+ transporting, mitochondrial Fo complex, subunit G |
| ATP5G3 | 207507_s_at | ATP synthase, H+ transporting, mitochondrial Fo complex, subunit C3 (subunit 9) |
| COX5B | 202343_x_at | cytochrome c oxidase subunit Vb |
| SDHC | 202004_x_at | succinate dehydrogenase complex, subunit C, integral membrane protein, 15kDa |
| COX6C | 201754_at | cytochrome c oxidase subunit VIc |
| NDUFA10 | 217860_at | NADH dehydrogenase (ubiquinone) 1 alpha subcomplex, 10, 42kDa |
| UQCRH | 202233_s_at | ubiquinol-cytochrome c reductase hinge protein |
| NDUFA3 | 218563_at | NADH dehydrogenase (ubiquinone) 1 alpha subcomplex, 3, 9kDa |
| NDUFB10 | 223112_s_at | NADH dehydrogenase (ubiquinone) 1 beta subcomplex, 10, 22kDa |
| NDUFS3 | 201740_at | NADH dehydrogenase (ubiquinone) Fe-S protein 3, 30kDa (NADH-coenzyme Q reductase) |
| UQCR10 | 218190_s_at | Homo sapiens ubiquinol-cytochrome c reductase, complex III subunit X |
| CYC1 | 201066_at | cytochrome c-1 |
| ATP5G1 | 208972_s_at | ATP synthase, H+ transporting, mitochondrial Fo complex, subunit C1 (subunit 9) |
| COX6A2 | 206353_at | cytochrome c oxidase subunit VIa polypeptide 2 |
| NDUFB3 | 203371_s_at | NADH dehydrogenase (ubiquinone) 1 beta subcomplex, 3, 12kDa |

| | | |
|---|---|---|
| | Mitochondrial Dysfunction | |
| PRDX5 | 222994_at | peroxiredoxin 5 |
| SDHA | 201093_x_at | succinate dehydrogenase complex, subunit A, flavoprotein (Fp) |
| MT-CO2 | 1553570_x_at | mitochondrially encoded cytochrome c oxidase II |
| MT-COI | 1553538_s_at | cytochrome c oxidase subunit I, Mitochondrion [Homo sapiens] |
| PRDX3 | 201619_at | peroxiredoxin 3 |
| NDUFA11 | 225304_s_at | NADH dehydrogenase (ubiquinone) 1 alpha subcomplex, 11, 14.7kDa |
| NDUFB7 | 202839_s_at | NADH dehydrogenase (ubiquinone) 1 beta subcomplex, 7, 18kDa |
| ATP5A1 | 213738_s_at | ATP synthase, H+ transporting, mitochondrial F1 complex, alpha subunit 1, cardiac muscle |

| | | |
|---|---|---|
| PARK7 | 200006_at | Parkinson disease (autosomal recessive, early onset) 7 |
| RHOT2 | 65770_at | ras homolog gene family, member T2 |
| NDUFS2 | 201966_at | NADH dehydrogenase (ubiquinone) Fe-S protein 2, 49kDa (NADH-coenzyme Q reductase) |
| TXN2 | 209078_s_at | thioredoxin 2 |
| NDUFB9 | 222992_s_at | NADH dehydrogenase (ubiquinone) 1 beta subcomplex, 9, 22kDa |
| COX7B | 202110_at | cytochrome c oxidase subunit VIIb |
| UQCRB | 205849_s_at | ubiquinol-cytochrome c reductase binding protein |
| NDUFS4 | 209303_at | NADH dehydrogenase (ubiquinone) Fe-S protein 4, 18kDa (NADH-coenzyme Q reductase) |
| NDUFA12 | 223244_s_at | NADH dehydrogenase (ubiquinone) 1 alpha subcomplex, 12 |
| ATP5B | 201322_at | ATP synthase, H+ transporting, mitochondrial F1 complex, beta polypeptide |
| UQCRFS1 | 208909_at | ubiquinol-cytochrome c reductase, Rieske iron-sulfur polypeptide 1 |
| NDUFS6 | 203606_at | NADH dehydrogenase (ubiquinone) Fe-S protein 6, 13kDa (NADH-coenzyme Q reductase) |
| COX4I1 | 200086_s_at | cytochrome c oxidase subunit IV isoform 1 |
| NDUFB4 | 218226_s_at | NADH dehydrogenase (ubiquinone) 1 beta subcomplex, 4, 15kDa |
| NDUFB8 | 201226_at | NADH dehydrogenase (ubiquinone) 1 beta subcomplex, 8, 19kDa |
| NDUFB6 | 203613_s_at | NADH dehydrogenase (ubiquinone) 1 beta subcomplex, 6, 17kDa |
| MAOB | 204041_at | monoamine oxidase B |
| NDUFA2 | 209224_s_at | NADH dehydrogenase (ubiquinone) 1 alpha subcomplex, 2, 8kDa |
| COX7C | 201134_x_at | cytochrome c oxidase subunit VIIc |
| NDUFB1 | 206790_s_at | NADH dehydrogenase (ubiquinone) 1 beta subcomplex, 1, 7kDa |
| NDUFA13 | 220864_s_at | NADH dehydrogenase (ubiquinone) 1 alpha subcomplex, 13 |
| UQCRC2 | 200883_at | ubiquinol-cytochrome c reductase core protein II |
| NDUFB5 | 203621_at | NADH dehydrogenase (ubiquinone) 1 beta subcomplex, 5, 16kDa |
| NDUFAB1 | 202077_at | NADH dehydrogenase (ubiquinone) 1, alpha/beta subcomplex, 1, 8kDa |
| NDUFA9 | 208969_at | NADH dehydrogenase (ubiquinone) 1 alpha subcomplex, 9, 39kDa |
| NDUFB2 | 218200_s_at | NADH dehydrogenase (ubiquinone) 1 beta subcomplex, 2, 8kDa |
| COX5A | 203663_s_at | cytochrome c oxidase subunit Va |
| NDUFS8 | 203189_s_at | NADH dehydrogenase (ubiquinone) Fe-S protein 8, 23kDa (NADH-coenzyme Q reductase) |
| PDHA1 | 200980_s_at | pyruvate dehydrogenase (lipoamide) alpha 1 |
| NDUFA8 | 218160_at | NADH dehydrogenase (ubiquinone) 1 alpha subcomplex, 8, 19kDa |
| NDUFS7 | 211752_s_at | NADH dehydrogenase (ubiquinone) Fe-S protein 7, 20kDa (NADH-coenzyme Q reductase) |
| SDHB | 202675_at | succinate dehydrogenase complex, subunit B, iron sulfur (Ip) |
| COX8A | 201119_s_at | cytochrome c oxidase subunit VIIIA (ubiquitous) |
| COX6B1 | 201441_at | cytochrome c oxidase subunit VIb polypeptide 1 (ubiquitous) |
| NDUFB11 | 218320_s_at | NADH dehydrogenase (ubiquinone) 1 beta subcomplex, 11, 17.3kDa |
| NDUFV1 | 208714_at | NADH dehydrogenase (ubiquinone) flavoprotein 1, 51kDa |
| CYCS | 208905_at | cytochrome c, somatic |
| CPT1B | 210069_at | carnitine palmitoyltransferase 1B (muscle) |
| COX5B | 202343_x_at | cytochrome c oxidase subunit Vb |
| SDHC | 202004_x_at | succinate dehydrogenase complex, subunit C, integral membrane protein, 15kDa |
| COX6C | 201754_at | cytochrome c oxidase subunit VIc |

| | | |
|---|---|---|
| NDUFA10 | 217860_at | NADH dehydrogenase (ubiquinone) 1 alpha subcomplex, 10, 42kDa |
| UQCRH | 202233_s_at | ubiquinol-cytochrome c reductase hinge protein |
| NDUFA3 | 218563_at | NADH dehydrogenase (ubiquinone) 1 alpha subcomplex, 3, 9kDa |
| NDUFB10 | 223112_s_at | NADH dehydrogenase (ubiquinone) 1 beta subcomplex, 10, 22kDa |
| NDUFS3 | 201740_at | NADH dehydrogenase (ubiquinone) Fe-S protein 3, 30kDa (NADH-coenzyme Q reductase) |
| PINK1 | 209018_s_at | PTEN induced putative kinase 1 |
| CYC1 | 201066_at | cytochrome c-1 |
| COX6A2 | 206353_at | cytochrome c oxidase subunit VIa polypeptide 2 |
| NDUFB3 | 203371_s_at | NADH dehydrogenase (ubiquinone) 1 beta subcomplex, 3, 12kDa |
| | | |
| | Ubiquinone Biosynthesis | |
| UFSP2 | 218449_at | UFM1-specific peptidase 2 |
| NDUFC1 | 203478_at | NADH dehydrogenase (ubiquinone) 1, subcomplex unknown, 1, 6kDa |
| | | |
| NDUFA11 | 225304_s_at | NADH dehydrogenase (ubiquinone) 1 alpha subcomplex, 11, 14.7kDa |
| NDUFB7 | 202839_s_at | NADH dehydrogenase (ubiquinone) 1 beta subcomplex, 7, 18kDa |
| NDUFS2 | 201966_at | NADH dehydrogenase (ubiquinone) Fe-S protein 2, 49kDa (NADH-coenzyme Q reductase) |
| NDUFB9 | 222992_s_at | NADH dehydrogenase (ubiquinone) 1 beta subcomplex, 9, 22kDa |
| MGMT | 204880_at | Homo sapiens O-6-methylguanine-DNA methyltransferase |
| NDUFC2 | 218101_s_at | NADH dehydrogenase (ubiquinone) 1, subcomplex unknown, 2, 14.5kDa |
| NDUFS4 | 209303_at | NADH dehydrogenase (ubiquinone) Fe-S protein 4, 18kDa (NADH-coenzyme Q reductase) |
| | | |
| NDUFA12 | 223244_s_at | NADH dehydrogenase (ubiquinone) 1 alpha subcomplex, 12 |
| NDUFS6 | 203606_at | NADH dehydrogenase (ubiquinone) Fe-S protein 6, 13kDa (NADH-coenzyme Q reductase) |
| NDUFB4 | 218226_s_at | NADH dehydrogenase (ubiquinone) 1 beta subcomplex, 4, 15kDa |
| NDUFB8 | 201226_at | NADH dehydrogenase (ubiquinone) 1 beta subcomplex, 8, 19kDa |
| NDUFB6 | 203613_s_at | NADH dehydrogenase (ubiquinone) 1 beta subcomplex, 6, 17kDa |
| NDUFA2 | 209224_s_at | NADH dehydrogenase (ubiquinone) 1 alpha subcomplex, 2, 8kDa |
| NDUFB1 | 206790_s_at | NADH dehydrogenase (ubiquinone) 1 beta subcomplex, 1, 7kDa |
| | | |
| NDUFA13 | 220864_s_at | NADH dehydrogenase (ubiquinone) 1 alpha subcomplex, 13 |
| NDUFB5 | 203621_at | NADH dehydrogenase (ubiquinone) 1 beta subcomplex, 5, 16kDa |
| | | |
| NDUFAB1 | 202077_at | NADH dehydrogenase (ubiquinone) 1, alpha/beta subcomplex, 1, 8kDa |
| NDUFA9 | 208969_at | NADH dehydrogenase (ubiquinone) 1 alpha subcomplex, 9, 39kDa |
| NDUFB2 | 218200_s_at | NADH dehydrogenase (ubiquinone) 1 beta subcomplex, 2, 8kDa |
| NDUFA1 | 202298_at | NADH dehydrogenase (ubiquinone) 1 alpha subcomplex, 1, 7.5kDa |
| NDUFS8 | 203189_s_at | NADH dehydrogenase (ubiquinone) Fe-S protein 8, 23kDa (NADH-coenzyme Q reductase) |
| NDUFA8 | 218160_at | NADH dehydrogenase (ubiquinone) 1 alpha subcomplex, 8, 19kDa |
| NDUFS7 | 211752_s_at | NADH dehydrogenase (ubiquinone) Fe-S protein 7, 20kDa (NADH-coenzyme Q reductase) |
| NDUFB11 | 218320_s_at | NADH dehydrogenase (ubiquinone) 1 beta subcomplex, 11, 17.3kDa |
| NDUFV1 | 208714_at | NADH dehydrogenase (ubiquinone) flavoprotein 1, 51kDa |
| | 217860_at | NADH dehydrogenase (ubiquinone) 1 alpha subcomplex, 10, 42kDa |

NDUFA10

| | | |
|---|---|---|
| NDUFA3 | 218563_at | NADH dehydrogenase (ubiquinone) 1 alpha subcomplex, 3, 9kDa |
| NDUFB10 | 223112_s_at | NADH dehydrogenase (ubiquinone) 1 beta subcomplex, 10, 22kDa |
| NDUFS3 | 201740_at | NADH dehydrogenase (ubiquinone) Fe-S protein 3, 30kDa (NADH-coenzyme Q reductase) |
| BCKDHB | 210653_s_at | branched chain keto acid dehydrogenase E1, beta polypeptide |
| NDUFB3 | 203371_s_at | NADH dehydrogenase (ubiquinone) 1 beta subcomplex, 3, 12kDa |

Protein Ubiquitination Pathway

| | | |
|---|---|---|
| VHL | 203844_at | von Hippel-Lindau tumor suppressor |
| CBL | 229010_at | Cas-Br-M (murine) ecotropic retroviral transforming sequence |
| USP36 | 220370_s_at | ubiquitin specific peptidase 36 |
| DNAJC6 | 204720_s_at | DnaJ (Hsp40) homolog, subfamily C, member 6 |
| USP51 | 237247_at | ubiquitin specific peptidase 51 |
| DNAJC21 | 230893_at | DnaJ (Hsp40) homolog, subfamily C, member 21 |
| USP12 | 213327_s_at | ubiquitin specific peptidase 12 |
| MDM2 | 225160_x_at | Mdm2 p53 binding protein homolog (mouse) |
| PSMD5 | 203447_at | proteasome (prosome, macropain) 26S subunit, non-ATPase, 5 |
| NEDD4L | 212448_at | neural precursor cell expressed, developmentally down-regulated 4-like |
| HLA-C | 216526_x_at | major histocompatibility complex, class I, C |
| USP45 | 238057_at | ubiquitin specific peptidase 45 |
| HSPA13 | 202558_s_at | heat shock protein 70kDa family, member 13 |
| HSPA14 | 226887_at | heat shock 70kDa protein 14 |
| USP1 | 202412_s_at | ubiquitin specific peptidase 1 |
| USP34 | 212065_s_at | ubiquitin specific peptidase 34 |
| HSPB8 | 233057_at | heat shock 22kDa protein 8 |
| USP54 | 227754_at | Homo sapiens ubiquitin specific peptidase 54 |
| USP42 | 226176_s_at | ubiquitin specific peptidase 42 |
| USP33 | 212513_s_at | ubiquitin specific peptidase 33 |
| SMURF2 | 205596_s_at | SMAD specific E3 ubiquitin protein ligase 2 |
| DNAJB14 | 222850_s_at | DnaJ (Hsp40) homolog, subfamily B, member 14 |
| DNAJC13 | 212467_at | DnaJ (Hsp40) homolog, subfamily C, member 13 |
| XIAP | 225859_at | X-linked inhibitor of apoptosis |
| DNAJB1 | 200666_s_at | DnaJ (Hsp40) homolog, subfamily B, member 1 |
| USP9X | 201100_s_at | ubiquitin specific peptidase 9, X-linked |
| USP8 | 202745_at | ubiquitin specific peptidase 8 |
| BIRC6 | 224635_s_at | baculoviral IAP repeat containing 6 |
| UBE2G2 | 1557053_s_at | ubiquitin-conjugating enzyme E2G 2 (UBC7 homolog, yeast) |
| CUL2 | 203079_s_at | cullin 2 |
| PSMD13 | 201232_s_at | proteasome (prosome, macropain) 26S subunit, non-ATPase, 13 |
| PSMD6 | 202753_at | proteasome (prosome, macropain) 26S subunit, non-ATPase, 6 |
| ANAPC5 | 200098_s_at | anaphase promoting complex subunit 5 |
| PSMB1 | 200876_s_at | proteasome (prosome, macropain) subunit, beta type, 1 |
| CDC34 | 212540_at | cell division cycle 34 homolog (S. cerevisiae) |
| PSMD14 | 212296_at | proteasome (prosome, macropain) 26S subunit, non-ATPase, 14 |
| UBE2D3 | 200668_s_at | ubiquitin-conjugating enzyme E2D 3 (UBC4/5 homolog, yeast) |
| PSMB4 | 202243_s_at | proteasome (prosome, macropain) subunit, beta type, 4 |

| | | |
|---|---|---|
| UBE2N | 201523_x_at | ubiquitin-conjugating enzyme E2N (UBC13 homolog, yeast) |
| UBB | 200633_at | ubiquitin B |
| TCEB2 | 200085_s_at | transcription elongation factor B (SIII), polypeptide 2 (18kDa, elongin B) |
| PSMA5 | 201274_at | proteasome (prosome, macropain) subunit, alpha type, 5 |
| PSMA1 | 210759_s_at | proteasome (prosome, macropain) subunit, alpha type, 1 |
| STUB1 | 217934_x_at | |
| RBX1 | 218117_at | ring-box 1, E3 ubiquitin protein ligase |
| USP2 | 229337_at | ubiquitin specific peptidase 2 |
| UBE2A | 201898_s_at | ubiquitin-conjugating enzyme E2A (RAD6 homolog) |
| CRYAB | 209283_at | crystallin, alpha B |
| DNAJC25 | 226859_at | DnaJ (Hsp40) homolog, subfamily C , member 25 |
| USP21 | 218367_x_at | ubiquitin specific peptidase 21 |
| PSMD9 | 207805_s_at | proteasome (prosome, macropain) 26S subunit, non-ATPase, 9 |
| TCEB1 | 202824_s_at | transcription elongation factor B (SIII), polypeptide 1 (15kDa, elongin C) |
| DNAJC1 | 218409_s_at | proteasome (prosome, macropain) subunit, beta type, 6 |
| PSMB6 | 208827_at | |
| DNAJC19 | 225358_at | DnaJ (Hsp40) homolog, subfamily C, member 19 |
| UBE2D4 | 221949_at | Homo sapiens ubiquitin-conjugating enzyme E2D 4 |
| PSMB10 | 202659_at | proteasome (prosome, macropain) subunit, beta type, 10 |
| BAG1 | 202387_at | BCL2-associated athanogene |
| THOP1 | 203235_at | thimet oligopeptidase 1 |
| DNAJC17 | 219861_at | DnaJ (Hsp40) homolog, subfamily C, member 17 |
| DNAJC12 | 223721_s_at | DnaJ (Hsp40) homolog, subfamily C, member 12 |
| DNAJC28 | 220372_at | DnaJ (Hsp40) homolog, subfamily C, member 28 |
| ANAPC11 | 226414_s_at | anaphase promoting complex subunit 11 |
| DNAJC14 | 1554451_s_at | DnaJ (Hsp40) homolog, subfamily C, member 14 |

| | BMP signaling pathway | |
|---|---|---|
| FST | 226847_at | follistatin |
| NOG | 231798_at | noggin |
| NFKB2 | 209636_at | nuclear factor of kappa light polypeptide gene enhancer in B-cells 2 (p49/p100) |
| SMAD7 | 204790_at | SMAD family member 7 |
| BMP2 | 205289_at | bone morphogenetic protein 2 |
| SMAD1 | 208015_at | SMAD family member 1 |
| SMAD9 | 227719_at | SMAD family member 9 |
| SMAD5 | 205187_at | SMAD family member 5 |
| BMP5 | 205430_at | bone morphogenetic protein 5 |
| PRKAR1A | 200605_s_at | protein kinase, cAMP-dependent, regulatory, type I, alpha (tissue specific extinguisher 1) |
| JUN | 201465_s_at | jun proto-oncogene |
| ATF2 | 212984_at | activating transcription factor 2 |
| KRAS | 204009_s_at | v-Ki-ras2 Kirsten rat sarcoma viral oncogene homolog |
| SOS1 | 227426_at | son of sevenless homolog 1 (Drosophila) |
| CREB1 | 204313_s_at | cAMP responsive element binding protein 1 |
| MRAS | 225185_at | muscle RAS oncogene homolog |
| MAPK1 | 224621_at | mitogen-activated protein kinase 1 |
| CREBBP | 202160_at | CREB binding protein |

| | | |
|---|---|---|
| XIAP | 225859_at | X-linked inhibitor of apoptosis |
| BMPR2 | 225144_at | bone morphogenetic protein receptor, type II (serine/threonine kinase) |
| NRAS | 202647_s_at | neuroblastoma RAS viral (v-ras) oncogene homolog |
| BMPR1A | 230979_at | bone morphogenetic protein receptor, type IA |
| MAPK3 | 212046_x_at | mitogen-activated protein kinase 3 |
| MAP2K2 | 202424_at | mitogen-activated protein kinase kinase 2 |
| PRKAG1 | 201805_at | protein kinase, AMP-activated, gamma 1 non-catalytic subunit |

**3 wks rehab vs. control**

| Gene | Affymetrix accession # | Gene description |
|---|---|---|
| | Protein Ubiquitination Pathway | |
| HSPA6 | 213418_at | heat shock 70kDa protein 6 (HSP70B') |
| USP27X | 217605_at | ubiquitin specific peptidase 27, X-linked |
| UBE2C | 202954_at | ubiquitin-conjugating enzyme E2C |
| USP36 | 220370_s_at | ubiquitin specific peptidase 36 |
| B2M | 232311_at | beta-2-microglobulin |
| CBL | 225231_at | Cas-Br-M (murine) ecotropic retroviral transforming sequence |
| HLA-B | 208729_x_at | major histocompatibility complex, class I, B |
| NEDD4L | 212445_s_at | neural precursor cell expressed, developmentally down-regulated 4-like |
| USP9X | 229573_at | ubiquitin specific peptidase 9, X-linked |
| HLA-C | 216526_x_at | major histocompatibility complex, class I, C |
| SMURF2 | 227489_at | SMAD specific E3 ubiquitin protein ligase 2 |
| HLA-A | 215313_x_at | Homo sapiens major histocompatibility complex, class I, A ( |
| HSPA12B | 229172_at | heat shock 70kD protein 12B |
| HSPB11 | 203960_s_at | heat shock protein family B (small), member 11 |
| HSPA14 | 226887_at | heat shock 70kDa protein 14 |
| USP33 | 212513_s_at | ubiquitin specific peptidase 33 |
| DNAJB14 | 222850_s_at | DnaJ (Hsp40) homolog, subfamily B, member 14 |
| HSPA12A | 214434_at | heat shock 70kDa protein 12A |
| USP1 | 202413_s_at | ubiquitin specific peptidase 1 |
| UBE2V1 | 201003_x_at | TMEM189-UBE2V1 readthrough |
| USP48 | 220079_s_at | ubiquitin specific peptidase 48 |
| SUGT1 | 223329_x_at | SGT1, suppressor of G2 allele of SKP1 (S. cerevisiae) |
| UBA1 | 200964_at | ubiquitin-like modifier activating enzyme 1 |
| USP47 | 221518_s_at | ubiquitin specific peptidase 47 |
| XIAP | 235222_x_at | Homo sapiens X-linked inhibitor of apoptosis |
| PSMC5 | 209503_s_at | proteasome (prosome, macropain) 26S subunit, ATPase, 5 |
| DNAJC24 | 213853_at | DnaJ (Hsp40) homolog, subfamily C, member 24 |
| PSMB1 | 200876_s_at | proteasome (prosome, macropain) subunit, beta type, 1 |
| DNAJB9 | 202842_s_at | DnaJ (Hsp40) homolog, subfamily B, member 9 |
| PSMD2 | 200830_at | proteasome (prosome, macropain) 26S subunit, non-ATPase, 2 |
| UBE3B | 212403_at | ubiquitin protein ligase E3B |
| PSMC1 | 204219_s_at | proteasome (prosome, macropain) 26S subunit, ATPase, 1 |
| CUL2 | 203079_s_at | cullin 2 |
| PSMB4 | 202243_s_at | proteasome (prosome, macropain) subunit, beta type, 4 |
| PSMA6 | 208805_at | proteasome (prosome, macropain) subunit, alpha type, 6 |
| PSMA2 | 201317_s_at | proteasome (prosome, macropain) subunit, alpha type, 2 |
| DNAJB2 | 202500_at | DnaJ (Hsp40) homolog, subfamily B, member 2 |
| AMFR | 202204_s_at | Homo sapiens autocrine motility factor receptor |
| CDC34 | 212540_at | cell division cycle 34 homolog (S. cerevisiae) |
| CRYAB | 209283_at | crystallin, alpha B |
| UBE2D3 | 200668_s_at | ubiquitin-conjugating enzyme E2D 3 (UBC4/5 homolog, yeast) |
| PSMD4 | 211609_x_at | proteasome (prosome, macropain) 26S subunit, non-ATPase, 4 |
| PSMD6 | 202753_at | proteasome (prosome, macropain) 26S subunit, non-ATPase, 6 |

| | | |
|---|---|---|
| PSMB2 | 200039_s_at | proteasome (prosome, macropain) subunit, beta type, 2 |
| BAG1 | 202387_at | BCL2-associated athanogene |
| PSMA1 | 201676_x_at | proteasome (prosome, macropain) subunit, alpha type, 1 |
| UBE2N | 201523_x_at | ubiquitin-conjugating enzyme E2N (UBC13 homolog, yeast) |
| PSMD13 | 201232_s_at | proteasome (prosome, macropain) 26S subunit, non-ATPase, 13 |
| USP13 | 227788_at | ubiquitin specific peptidase 13 (isopeptidase T-3) |
| PSMC2 | 201067_at | proteasome (prosome, macropain) 26S subunit, ATPase, 2 |
| PSMB5 | 208799_at | proteasome (prosome, macropain) subunit, beta type, 5 |
| PSMD12 | 202352_s_at | proteasome (prosome, macropain) 26S subunit, non-ATPase, 12 |
| PSMA7 | 201114_x_at | proteasome (prosome, macropain) subunit, alpha type, 7 |
| PSMD1 | 201198_s_at | proteasome (prosome, macropain) 26S subunit, non-ATPase, 1 |
| PSMD14 | 212296_at | proteasome (prosome, macropain) 26S subunit, non-ATPase, 14 |
| PSMA3 | 201532_at | proteasome (prosome, macropain) subunit, alpha type, 3 |
| UBE2M | 203109_at | Homo sapiens ubiquitin-conjugating enzyme E2M |
| UBE2F | 225783_at | ubiquitin-conjugating enzyme E2F (putative) |
| PSMA5 | 201274_at | proteasome (prosome, macropain) subunit, alpha type, 5 |
| HSP90AA1 | 210211_s_at | heat shock protein 90kDa alpha (cytosolic), class A member 1 |
| TCEB1 | 202824_s_at | transcription elongation factor B (SIII), polypeptide 1 (15kDa, elongin C) |
| DNAJC8 | 212490_at | DnaJ (Hsp40) homolog, subfamily C, member 8 |
| HSPE1 | 205133_s_at | Homo sapiens heat shock 10kDa protein 1 |
| PSMD11 | 208777_s_at | proteasome (prosome, macropain) 26S subunit, non-ATPase, 11 |
| DNAJC11 | 215792_s_at | DnaJ (Hsp40) homolog, subfamily C, member 11 |
| HSPD1 | 200806_s_at | Homo sapiens heat shock 60kDa protein 1 |
| PSMB6 | 208827_at | proteasome (prosome, macropain) subunit, beta type, 6 |
| HSP90AB1 | 214359_s_at | Homo sapiens heat shock protein 90kDa alpha (cytosolic), class B member 1 |
| THOP1 | 203235_at | thimet oligopeptidase 1 |
| UBB | 217125_at | Homo sapiens ubiquitin B |
| PSMD3 | 201388_at | proteasome (prosome, macropain) 26S subunit, non-ATPase, 3 |
| HSPA1A/HSPA1B | 200799_at | Homo sapiens heat shock 70kDa protein 1A |
| PSMD8 | 200820_at | proteasome (prosome, macropain) 26S subunit, non-ATPase, 8 |
| USP24 | 212381_at | ubiquitin specific peptidase 24 |
| USP54 | 227334_at | ubiquitin specific peptidase 54 |
| UBE2L3 | 200676_s_at | ubiquitin-conjugating enzyme E2L 3 |
| HSPA8 | 208687_x_at | heat shock 70kDa protein 8 |
| HSPH1 | 208744_x_at | Homo sapiens HSP105 alpha |
| USP3 | 201536_at | ubiquitin specific peptidase 3 |
| DNAJB6 | 209015_s_at | DnaJ (Hsp40) homolog, subfamily B, member 6 |
| DNAJB4 | 203811_s_at | DnaJ (Hsp40) homolog, subfamily B, member 4 |
| DNAJC28 | 220372_at | DnaJ (Hsp40) homolog, subfamily C, member 28 |
| HSPA9 | 200690_at | heat shock 70kDa protein 9 (mortalin) |
| DNAJC14 | 1554451_s_at | DnaJ (Hsp40) homolog, subfamily C, member 14 |
| | Fc¦Ã Receptor-mediated Phagocytosis in Macrophages and Monocytes | |
| ACTA2 | 243140_at | actin, alpha 2, smooth muscle, aorta |
| FCGR2A | 203561_at | Fc fragment of IgG, low affinity IIa, receptor (CD32) |
| VAV2 | 226063_at | vav 2 guanine nucleotide exchange factor |
| LYN | 202625_at | v-yes-1 Yamaguchi sarcoma viral related oncogene homolog |

| | | |
|---|---|---|
| AKT2 | 203809_s_at | v-akt murine thymoma viral oncogene homolog 2 |
| LCP2 | 205270_s_at | lymphocyte cytosolic protein 2 (SH2 domain containing leukocyte protein of 76 |
| AKT3 | 212609_s_at | v-akt murine thymoma viral oncogene homolog 3 (protein kinase B, gamma) |
| SYK | 226068_at | spleen tyrosine kinase |
| HCK | 208018_s_at | hemopoietic cell kinase |
| RAC2 | 213603_s_at | ras-related C3 botulinum toxin substrate 2 (rho family, small GTP binding prote |
| INPP5D | 203332_s_at | inositol polyphosphate-5-phosphatase, 145kDa |
| PLD1 | 226636_at | phospholipase D1, phosphatidylcholine-specific |
| FYN | 210105_s_at | FYN oncogene related to SRC, FGR, YES |
| FYB | 211795_s_at | FYN binding protein |
| ARPC5 | 211963_s_at | actin related protein 2/3 complex, subunit 5, 16kDa |
| EZR | 217234_s_at | ezrin |
| CBL | 225231_at | Cas-Br-M (murine) ecotropic retroviral transforming sequence |
| PAK1 | 226507_at | p21 protein (Cdc42/Rac)-activated kinase 1 |
| PLCG1 | 202789_at | phospholipase C, gamma 1 |
| MYO5A | 227761_at | myosin VA (heavy chain 12, myoxin) |
| YES1 | 202932_at | v-yes-1 Yamaguchi sarcoma viral oncogene homolog 1 |
| ACTG1 | 201550_x_at | actin, gamma 1 |
| PXN | 201087_at | paxillin |
| PTEN | 233314_at | phosphatase and tensin homolog |
| PRKCI | 213518_at | protein kinase C, iota |
| VAMP3 | 211749_s_at | vesicle-associated membrane protein 3 (cellubrevin) |
| PIP5K1A | 207391_s_at | phosphatidylinositol-4-phosphate 5-kinase, type I, alpha |
| ACTB | AFFX-HSAC07/X00351_5_at | actin, beta |
| ARF6 | 203311_s_at | ADP-ribosylation factor 6 |
| MAPK1 | 208351_s_at | mitogen-activated protein kinase 1 |
| RAB11B | 217793_at | RAB11B, member RAS oncogene family |
| | | |
| | ILK Signaling | |
| MYL5 | 205145_s_at | myosin, light chain 5, regulatory |
| MYH8 | 206717_at | myosin, heavy chain 8, skeletal muscle, perinatal |
| MYH1 | 205951_at | myosin, heavy chain 1, skeletal muscle, adult |
| ACTA2 | 243140_at | actin, alpha 2, smooth muscle, aorta |
| ACTN3 | 206891_at | actinin, alpha 3 |
| ITGB8 | 226189_at | integrin, beta 8 |
| MYH13 | 208208_at | myosin, heavy chain 13, skeletal muscle |
| PARVA | 222454_s_at | parvin, alpha |
| FBLIM1 | 225258_at | filamin binding LIM protein 1 |
| MYH4 | 208148_at | myosin, heavy chain 4, skeletal muscle |
| TGFB1I1 | 209651_at | transforming growth factor beta 1 induced transcript 1 |
| PIK3CD | 203879_at | phosphoinositide-3-kinase, catalytic, delta polypeptide |
| AKT2 | 203809_s_at | v-akt murine thymoma viral oncogene homolog 2 |
| BMP2 | 205289_at | bone morphogenetic protein 2 |
| ITGB2 | 202803_s_at | integrin, beta 2 (complement component 3 receptor 3 and 4 subunit) |
| MYL6 | 214002_at | Homo sapiens myosin, light chain 6, alkali, smooth muscle and non-muscle |
| AKT3 | 212609_s_at | v-akt murine thymoma viral oncogene homolog 3 (protein kinase B, gamma) |

| | | |
|---|---|---|
| VIM | 201426_s_at | vimentin |
| FN1 | 211719_x_at | fibronectin 1 |
| RHOB | 212099_at | ras homolog gene family, member B |
| TMSB10/TMSB4X | 217733_s_at | thymosin beta 10 |
| PTK2 | 1559529_at | PTK2 protein tyrosine kinase 2 |
| CTNNB1 | 223679_at | catenin (cadherin-associated protein), beta 1, 88kDa |
| RHOJ | 243481_at | ras homolog gene family, member J |
| PDGFC | 218718_at | platelet derived growth factor C |
| HIF1A | 200989_at | hypoxia inducible factor 1, alpha subunit (basic helix-loop-helix transcription fa |
| ACTG1 | 201550_x_at | actin, gamma 1 |
| ITGB5 | 201124_at | integrin, beta 5 |
| PXN | 201087_at | paxillin |
| PTEN | 233314_at | phosphatase and tensin homolog |
| PIK3C2A | 241905_at | phosphoinositide-3-kinase, class 2, alpha polypeptide |
| CHD1 | 204258_at | chromodomain helicase DNA binding protein 1 |
| ITGB1 | 211945_s_at | integrin, beta 1 (fibronectin receptor, beta polypeptide, antigen CD29 includes MSK12) |
| PPP2R5C | 1557718_at | protein phosphatase 2, regulatory subunit B', gamma |
| RHOT2 | 65770_at | ras homolog gene family, member T2 |
| SH2B2 | 205367_at | SH2B adaptor protein 2 |
| ACTB | AFFX-HSAC07/X00351_5_at | actin, beta |
| PPP2R2A | 202313_at | protein phosphatase 2, regulatory subunit B, alpha |
| ACTN2 | 203861_s_at | actinin, alpha 2 |
| MAPK1 | 208351_s_at | mitogen-activated protein kinase 1 |
| CFL2 | 224352_s_at | cofilin 2 (muscle) |
| TESK1 | 204106_at | testis-specific kinase 1 |
| PPP2R1A | 200695_at | protein phosphatase 2, regulatory subunit A, alpha |
| MYH7B | 233949_s_at | myosin, heavy chain 7B, cardiac muscle, beta |
| PPP2R5B | 635_s_at | protein phosphatase 2, regulatory subunit B', beta |
| MYH14 | 226988_s_at | myosin, heavy chain 14, non-muscle |
| MAP2K6 | 205698_s_at | mitogen-activated protein kinase kinase 6 |
| FLNC | 207876_s_at | filamin C, gamma |
| VEGFA | 210513_s_at | vascular endothelial growth factor A |
| PARVB | 216254_at | parvin, beta |
| | | |
| | Integrin Signaling | |
| MYL5 | 205145_s_at | myosin, light chain 5, regulatory |
| CAPN6 | 202965_s_at | calpain 6 |
| ACTA2 | 243140_at | actin, alpha 2, smooth muscle, aorta |
| TSPAN2 | 214606_at | tetraspanin 2 |
| ACTN3 | 206891_at | actinin, alpha 3 |
| ITGB8 | 226189_at | integrin, beta 8 |
| ITGA4 | 213416_at | integrin, alpha 4 (antigen CD49D, alpha 4 subunit of VLA-4 receptor) |
| PARVA | 222454_s_at | parvin, alpha |
| PIK3CD | 203879_at | phosphoinositide-3-kinase, catalytic, delta polypeptide |
| AKT2 | 203809_s_at | v-akt murine thymoma viral oncogene homolog 2 |
| ITGB2 | 202803_s_at | integrin, beta 2 (complement component 3 receptor 3 and 4 subunit) |

| | | |
|---|---|---|
| AKT3 | 212609_s_at | v-akt murine thymoma viral oncogene homolog 3 (protein kinase B, gamma) |
| RAC2 | 213603_s_at | ras-related C3 botulinum toxin substrate 2 (rho family, small GTP binding prote |
| RAP2B | 213923_at | RAP2B, member of RAS oncogene family |
| ITGA9 | 227297_at | integrin, alpha 9 |
| RHOB | 212099_at | ras homolog gene family, member B |
| WIPF1 | 202663_at | WAS/WASL interacting protein family, member 1 |
| PTK2 | 1559529_at | PTK2 protein tyrosine kinase 2 |
| FYN | 210105_s_at | FYN oncogene related to SRC, FGR, YES |
| ARPC5 | 211963_s_at | actin related protein 2/3 complex, subunit 5, 16kDa |
| RHOJ | 243481_at | ras homolog gene family, member J |
| PAK1 | 226507_at | p21 protein (Cdc42/Rac)-activated kinase 1 |
| ASAP1 | 224791_at | ArfGAP with SH3 domain, ankyrin repeat and PH domain 1 |
| PLCG1 | 202789_at | phospholipase C, gamma 1 |
| LIMS1 | 1570259_at | LIM and senescent cell antigen-like domains 1 |
| CAV1 | 203065_s_at | caveolin 1, caveolae protein, 22kDa |
| ACTG1 | 201550_x_at | actin, gamma 1 |
| ITGB5 | 201124_at | integrin, beta 5 |
| PXN | 201087_at | paxillin |
| PTEN | 233314_at | phosphatase and tensin homolog |
| RAP1B | 200833_s_at | RAP1B, member of RAS oncogene family |
| SHC1 | 214853_s_at | SHC (Src homology 2 domain containing) transforming protein 1 |
| PIK3C2A | 241905_at | phosphoinositide-3-kinase, class 2, alpha polypeptide |
| ARHGAP26 | 226576_at | Rho GTPase activating protein 26 |
| ARHGAP5 | 217936_at | Rho GTPase activating protein 5 |
| RALB | 202100_at | v-ral simian leukemia viral oncogene homolog B (ras related; GTP binding prote |
| ARHGEF7 | 202548_s_at | Rho guanine nucleotide exchange factor (GEF) 7 |
| ITGA6 | 215177_s_at | integrin, alpha 6 |
| ITGB1 | 211945_s_at | integrin, beta 1 (fibronectin receptor, beta polypeptide, antigen CD29 includes MSK12) |
| ARF1 | 200065_s_at | ADP-ribosylation factor 1 |
| CAPN7 | 203356_at | calpain 7 |
| TSPAN7 | 202242_at | tetraspanin 7 |
| RHOT2 | 65770_at | ras homolog gene family, member T2 |
| ACTB | AFFX-HSAC07/X00351_5_at | actin, beta |
| ARF6 | 203311_s_at | ADP-ribosylation factor 6 |
| ACTN2 | 203861_s_at | actinin, alpha 2 |
| MAPK1 | 208351_s_at | mitogen-activated protein kinase 1 |
| MYL12A | 201319_at | myosin, light chain 12A, regulatory, non-sarcomeric |
| MRAS | 206538_at | muscle RAS oncogene homolog |
| BCAR3 | 204032_at | breast cancer anti-estrogen resistance 3 |
| PARVB | 216254_at | parvin, beta |
| | Antigen Presentation Pathway | |
| NLRC5 | 226474_at | NLR family, CARD domain containing 5 |
| HLA-DPB1 | 201137_s_at | major histocompatibility complex, class II, DP beta 1 |
| B2M | 232311_at | beta-2-microglobulin |
| HLA-DPA1 | 211990_at | major histocompatibility complex, class II, DP alpha 1 |

| | | |
|---|---|---|
| HLA-DRA | 208894_at | major histocompatibility complex, class II, DR alpha |
| HLA-B | 208729_x_at | major histocompatibility complex, class I, B |
| HLA-F | 204806_x_at | major histocompatibility complex, class I, F |
| HLA-DMA | 217478_s_at | major histocompatibility complex, class II, DM alpha |
| HLA-E | 200904_at | major histocompatibility complex, class I, E |
| HLA-C | 216526_x_at | major histocompatibility complex, class I, C |
| HLA-DMB | 203932_at | major histocompatibility complex, class II, DM beta |
| HLA-A | 215313_x_at | major histocompatibility complex, class I, A |
| HLA-G | 211528_x_at | major histocompatibility complex, class I, G |
| PSMB5 | 208799_at | proteasome (prosome, macropain) subunit, beta type, 5 |
| PSMB6 | 208827_at | proteasome (prosome, macropain) subunit, beta type, 6 |

**6 wks rehab vs. control**

| Gene | Affymetrix accession # | Gene description |
|---|---|---|
| | Molecular Mechanisms of Cancer | |
| GNA15 | 205349_at | guanine nucleotide binding protein (G protein), alpha 15 (Gq class) |
| CCND1 | 208712_at | cyclin D1 |
| SMAD7 | 204790_at | SMAD family member 7 |
| FAS | 204781_s_at | Fas (TNF receptor superfamily, member 6) |
| SMAD9 | 227719_at | SMAD family member 9 |
| PRKD1 | 205880_at | protein kinase D1 |
| IRS1 | 242979_at | insulin receptor substrate 1 |
| BMP2 | 205290_s_at | bone morphogenetic protein 2 |
| BID | 204493_at | BH3 interacting domain death agonist |
| BMP5 | 205430_at | bone morphogenetic protein 5 |
| RHOB | 212099_at | ras homolog gene family, member B |
| PTK2 | 1559529_at | PTK2 protein tyrosine kinase 2 |
| FYN | 210105_s_at | FYN oncogene related to SRC, FGR, YES |
| SYNGAP1 | 230297_x_at | synaptic Ras GTPase activating protein 1 |
| CTNNB1 | 223679_at | catenin (cadherin-associated protein), beta 1, 88kDa |
| MYC | 202431_s_at | v-myc myelocytomatosis viral oncogene homolog (avian) |
| CDK6 | 224847_at | cyclin-dependent kinase 6 |
| RALGDS | 209050_s_at | ral guanine nucleotide dissociation stimulator |
| TCF4 | 203753_at | transcription factor 4 |
| JUN | 201466_s_at | jun proto-oncogene |
| RAP2B | 213923_at | RAP2B, member of RAS oncogene family |
| RHOJ | 238905_at | ras homolog gene family, member J |
| GAB1 | 229114_at | GRB2-associated binding protein 1 |
| RHOC | 200885_at | ras homolog gene family, member C |
| ARHGEF2 | 207629_s_at | Rho/Rac guanine nucleotide exchange factor (GEF) 2 |
| FZD4 | 218665_at | frizzled homolog 4 (Drosophila) |
| TCF3 | 213811_x_at | transcription factor 3 (E2A immunoglobulin enhancer binding factors E12/E47) |
| HIF1A | 200989_at | hypoxia inducible factor 1, alpha subunit (basic helix-loop-helix transcription fact |
| TGFBR2 | 208944_at | transforming growth factor, beta receptor II (70/80kDa) |
| GNAI2 | 201040_at | guanine nucleotide binding protein (G protein), alpha inhibiting activity polypept |
| RAP1B | 200833_s_at | RAP1B, member of RAS oncogene family |
| CASP7 | 207181_s_at | caspase 7, apoptosis-related cysteine peptidase |
| SMAD1 | 210993_s_at | SMAD family member 1 |
| PLCB1 | 213222_at | phospholipase C, beta 1 (phosphoinositide-specific) |
| RBPJ | 207785_s_at | recombination signal binding protein for immunoglobulin kappa J region |
| GNAI3 | 201179_s_at | guanine nucleotide binding protein (G protein), alpha inhibiting activity polypept |
| FNBP1 | 212288_at | formin binding protein 1 |
| CDC42 | 208728_s_at | cell division cycle 42 (GTP binding protein, 25kDa) |
| LAMTOR3 | 217971_at | late endosomal/lysosomal adaptor, MAPK and MTOR activator 3 |
| MAP2K4 | 203266_s_at | mitogen-activated protein kinase kinase 4 |
| XIAP | 235222_x_at | X-linked inhibitor of apoptosis |
| RHOT2 | 221789_x_at | ras homolog gene family, member T2 |
| MAPK14 | 210449_x_at | mitogen-activated protein kinase 14 |

| | | |
|---|---|---|
| CASP9 | 210775_x_at | caspase 9, apoptosis-related cysteine peptidase |
| MAP2K2 | 202424_at | mitogen-activated protein kinase kinase 2 |
| LRP6 | 34697_at | low density lipoprotein receptor-related protein 6 |
| MAPK3 | 212046_x_at | mitogen-activated protein kinase 3 |
| CFLAR | 209508_x_at | CASP8 and FADD-like apoptosis regulator |
| CAMK2B | 213276_at | calcium/calmodulin-dependent protein kinase II beta |
| HIPK2 | 213763_at | homeodomain interacting protein kinase 2 |
| AKT2 | 236664_at | v-akt murine thymoma viral oncogene homolog 2 |
| PA2G4 | 216422_at | proliferation-associated 2G4, 38kDa |
| PRKAG1 | 201805_at | protein kinase, AMP-activated, gamma 1 non-catalytic subunit |
| PSEN1 | 207782_s_at | presenilin 1 |
| SMAD5 | 205188_s_at | SMAD family member 5 |
| JAK1 | 239695_at | Janus kinase 1 |
| GSK3B | 240562_at | Isoform 1 of Glycogen synthase kinase-3 beta |
| ARHGEF12 | 1566093_at | Rho guanine nucleotide exchange factor (GEF) 12 |

| | ILK Signaling | |
|---|---|---|
| MYL5 | 205145_s_at | myosin, light chain 5, regulatory |
| CCND1 | 208712_at | cyclin D1 |
| ACTA2 | 243140_at | actin, alpha 2, smooth muscle, aorta |
| PGF | 209652_s_at | placental growth factor |
| FN1 | 211719_x_at | |
| IRS1 | 242979_at | insulin receptor substrate 1 |
| BMP2 | 205290_s_at | bone morphogenetic protein 2 |
| TMSB10/TMSB4X | 217733_s_at | thymosin beta 10 |
| RHOB | 212099_at | ras homolog gene family, member B |
| PTK2 | 1559529_at | PTK2 protein tyrosine kinase 2 |
| VIM | 201426_s_at | vimentin |
| CTNNB1 | 223679_at | catenin (cadherin-associated protein), beta 1, 88kDa |
| PDGFC | 218718_at | platelet derived growth factor C |
| MYC | 202431_s_at | v-myc myelocytomatosis viral oncogene homolog (avian) |
| JUN | 201466_s_at | jun proto-oncogene |
| RHOJ | 238905_at | ras homolog gene family, member J |
| RHOC | 200885_at | ras homolog gene family, member C |
| ACTB | 213867_x_at | actin, beta |
| HIF1A | 200989_at | hypoxia inducible factor 1, alpha subunit (basic helix-loop-helix transcription fact |
| ACTG1 | 211983_x_at | actin, gamma 1 |
| PTEN | 233254_x_at | phosphatase and tensin homolog |
| PARVA | 1563458_at | parvin, alpha |
| FNBP1 | 212288_at | formin binding protein 1 |
| CDC42 | 208728_s_at | cell division cycle 42 (GTP binding protein, 25kDa) |
| MAP2K4 | 203266_s_at | mitogen-activated protein kinase kinase 4 |
| PPP2R5D | 202513_s_at | protein phosphatase 2, regulatory subunit B', delta |
| RHOT2 | 221789_x_at | ras homolog gene family, member T2 |
| PPP2R5B | 635_s_at | protein phosphatase 2, regulatory subunit B', beta |
| MAPK3 | 212046_x_at | mitogen-activated protein kinase 3 |

| | | |
|---|---|---|
| AKT2 | 236664_at | v-akt murine thymoma viral oncogene homolog 2 |
| ACTN2 | 203863_at | actinin, alpha 2 |
| PPP2R1A | 200695_at | protein phosphatase 2, regulatory subunit A, alpha |
| PPP2CA | 235502_at | Serine/threonine-protein phosphatase 2A catalytic subunit alpha isoform |
| GSK3B | 240562_at | Isoform 1 of Glycogen synthase kinase-3 beta |
| ITGB3 | 204627_s_at | integrin, beta 3 (platelet glycoprotein IIIa, antigen CD61) |
| | Clathrin-mediated Endocytosis Signaling | |
| USP9X | 230543_at | ubiquitin specific peptidase 9, X-linked |
| ACTA2 | 243140_at | actin, alpha 2, smooth muscle, aorta |
| IGF1 | 209540_at | insulin-like growth factor 1 (somatomedin C) |
| PGF | 209652_s_at | placental growth factor |
| PDGFD | 219304_s_at | platelet derived growth factor D |
| ARRB1 | 43511_s_at | arrestin, beta 1 |
| DAB2 | 201278_at | disabled homolog 2, mitogen-responsive phosphoprotein (Drosophila) |
| PDGFC | 218718_at | platelet derived growth factor C |
| ACTB | 213867_x_at | actin, beta |
| ARF6 | 224788_at | ADP-ribosylation factor 6 |
| ARPC5 | 1555797_a_at | actin related protein 2/3 complex, subunit 5, 16kDa |
| ACTG1 | 211983_x_at | actin, gamma 1 |
| NUMB | 230462_at | numb homolog (Drosophila) |
| MYO6 | 203216_s_at | myosin VI |
| CSNK2A2 | 224922_at | casein kinase 2, alpha prime polypeptide |
| RAB5C | 201140_s_at | RAB5C, member RAS oncogene family |
| CSNK2B | 231777_at | Isoform 1 of Lymphocyte antigen 6 complex locus protein G5b |
| SNX9 | 223027_at | sorting nexin 9 |
| CDC42 | 208728_s_at | cell division cycle 42 (GTP binding protein, 25kDa) |
| PPP3CB | 202432_at | protein phosphatase 3, catalytic subunit, beta isozyme |
| TSG101 | 201758_at | tumor susceptibility gene 101 |
| PPP3CC | 207000_s_at | protein phosphatase 3, catalytic subunit, gamma isozyme |
| SH3GLB1 | 209091_s_at | SH3-domain GRB2-like endophilin B1 |
| CLTCL1 | 205944_s_at | clathrin, heavy chain-like 1 |
| DNM2 | 202253_s_at | dynamin 2 |
| CSNK2A1 | 212075_s_at | casein kinase 2, alpha 1 polypeptide |
| ACTR2 | 200727_s_at | ARP2 actin-related protein 2 homolog (yeast) |
| EPS15 | 234278_at | epidermal growth factor receptor pathway substrate 15 |
| FGF1 | 208240_s_at | fibroblast growth factor 1 (acidic) |
| CLTC | 210498_at | clathrin, heavy chain (Hc) |
| ITGB3 | 204627_s_at | integrin, beta 3 (platelet glycoprotein IIIa, antigen CD61) |
| | Integrin Signaling | |
| CAPN6 | 202965_s_at | calpain 6 |
| MYL5 | 205145_s_at | myosin, light chain 5, regulatory |
| ACTA2 | 243140_at | actin, alpha 2, smooth muscle, aorta |
| TSPAN2 | 227236_at | tetraspanin 2 |
| ITGA6 | 201656_at | integrin, alpha 6 |
| ARF1 | 232175_at | ADP-ribosylation factor 1 |
| RHOB | 212099_at | ras homolog gene family, member B |

| | | |
|---|---|---|
| PTK2 | 1559529_at | PTK2 protein tyrosine kinase 2 |
| FYN | 210105_s_at | FYN oncogene related to SRC, FGR, YES |
| RAP2B | 213923_at | RAP2B, member of RAS oncogene family |
| RHOJ | 238905_at | ras homolog gene family, member J |
| PLCG1 | 202789_at | phospholipase C, gamma 1 |
| RHOC | 200885_at | ras homolog gene family, member C |
| WIPF1 | 202664_at | WAS/WASL interacting protein family, member 1 |
| ITGA10 | 206766_at | integrin, alpha 10 |
| ACTB | 213867_x_at | actin, beta |
| ARF6 | 224788_at | ADP-ribosylation factor 6 |
| TLN1 | 203254_s_at | talin 1 |
| ARPC5 | 1555797_a_at | actin related protein 2/3 complex, subunit 5, 16kDa |
| ACTG1 | 211983_x_at | actin, gamma 1 |
| RAP1B | 200833_s_at | RAP1B, member of RAS oncogene family |
| ASAP1 | 224791_at | ASAP1 intronic transcript 1 (non-protein coding) |
| PTEN | 233254_x_at | phosphatase and tensin homolog |
| PARVA | 1563458_at | parvin, alpha |
| FNBP1 | 212288_at | formin binding protein 1 |
| CDC42 | 208728_s_at | cell division cycle 42 (GTP binding protein, 25kDa) |
| MAP2K4 | 203266_s_at | mitogen-activated protein kinase kinase 4 |
| RHOT2 | 221789_x_at | ras homolog gene family, member T2 |
| MAP2K2 | 202424_at | mitogen-activated protein kinase kinase 2 |
| MAPK3 | 212046_x_at | mitogen-activated protein kinase 3 |
| ACTR2 | 200727_s_at | ARP2 actin-related protein 2 homolog (yeast) |
| AKT2 | 236664_at | v-akt murine thymoma viral oncogene homolog 2 |
| ACTN2 | 203863_at | actinin, alpha 2 |
| GSK3B | 240562_at | Isoform 1 of Glycogen synthase kinase-3 beta |
| ITGB3 | 204627_s_at | integrin, beta 3 (platelet glycoprotein IIIa, antigen CD61) |
| | Protein Ubiquitination Pathway | |
| USP9X | 230543_at | ubiquitin specific peptidase 9, X-linked |
| HSPA6 | 213418_at | heat shock 70kDa protein 6 (HSP70B') |
| PSMB9 | 1555853_at | proteasome (prosome, macropain) subunit, beta type, 9 (large multifunctional p |
| HSPA13 | 202558_s_at | heat shock protein 70kDa family, member 13 |
| NEDD4L | 212448_at | neural precursor cell expressed, developmentally down-regulated 4-like |
| VHL | 1559227_s_at | von Hippel-Lindau tumor suppressor |
| HSPB11 | 203960_s_at | heat shock protein family B (small), member 11 |
| DNAJC18 | 238115_at | Homo sapiens DnaJ (Hsp40) homolog, subfamily C, member 18 |
| PSMD10 | 1554577_a_at | proteasome (prosome, macropain) 26S subunit, non-ATPase, 10 |
| USP1 | 202413_s_at | ubiquitin specific peptidase 1 |
| CDC23 | 202892_at | cell division cycle 23 homolog (S. cerevisiae) |
| PSMB1 | 200876_s_at | proteasome (prosome, macropain) subunit, beta type, 1 |
| RBX1 | 218117_at | ring-box 1, E3 ubiquitin protein ligase |
| UBE2N | 201523_x_at | ubiquitin-conjugating enzyme E2N (UBC13 homolog, yeast) |
| PSMD6 | 202753_at | proteasome (prosome, macropain) 26S subunit, non-ATPase, 6 |
| PSMD12 | 202353_s_at | proteasome (prosome, macropain) 26S subunit, non-ATPase, 12 |
| UBE2A | 201898_s_at | ubiquitin-conjugating enzyme E2A (RAD6 homolog) |
| CUL2 | 203079_s_at | cullin 2 |

| | | |
|---|---|---|
| CDC34 | 212540_at | cell division cycle 34 homolog (S. cerevisiae) |
| PSMD9 | 207805_s_at | proteasome (prosome, macropain) 26S subunit, non-ATPase, 9 |
| UBA1 | 200964_at | ubiquitin-like modifier activating enzyme 1 |
| TCEB1 | 202824_s_at | transcription elongation factor B (SIII), polypeptide 1 (15kDa, elongin C) |
| PSMB2 | 200039_s_at | proteasome (prosome, macropain) subunit, beta type, 2 |
| DNAJC7 | 202416_at | DnaJ (Hsp40) homolog, subfamily C, member 7 |
| HSP90AB1 | 200064_at | heat shock protein 90kDa alpha (cytosolic), class B member 1 |
| PSMC2 | 201067_at | proteasome (prosome, macropain) 26S subunit, ATPase, 2 |
| PSMB5 | 208799_at | proteasome (prosome, macropain) subunit, beta type, 5 |
| XIAP | 235222_x_at | X-linked inhibitor of apoptosis |
| SUGT1 | 224309_s_at | SGT1, suppressor of G2 allele of SKP1 (S. cerevisiae) |
| UBE2M | 203109_at | ubiquitin-conjugating enzyme E2M (UBC12 homolog, yeast) |
| PSMD4 | 211609_x_at | proteasome (prosome, macropain) 26S subunit, non-ATPase, 4 |
| DNAJC24 | 213853_at | DnaJ (Hsp40) homolog, subfamily C, member 24 |
| PSMD3 | 201388_at | proteasome (prosome, macropain) 26S subunit, non-ATPase, 3 |
| USP2 | 207213_s_at | ubiquitin specific peptidase 2 |
| HSPA9 | 200690_at | heat shock 70kDa protein 9 (mortalin) |
| USP16 | 222616_s_at | ubiquitin specific peptidase 16 |
| PSMD1 | 201198_s_at | proteasome (prosome, macropain) 26S subunit, non-ATPase, 1 |
| DNAJB6 | 209015_s_at | DnaJ (Hsp40) homolog, subfamily B, member 6 |
| USP37 | 232033_at | ubiquitin specific peptidase 37 |
| DNAJC28 | 220372_at | DnaJ (Hsp40) homolog, subfamily C, member 28 |
| BAG1 | 202387_at | BCL2-associated athanogene |
| UBR2 | 215558_at | ubiquitin protein ligase E3 component n-recognin 2 |
| USP54 | 227334_at | ubiquitin specific peptidase 54 |
