# Supplementary material for: Molecular signatures of differential responses to exercise trainings during rehabilitation
Source: Biomed Genet Genom. Author manuscript; Available in PMC 2017 Aug 23. (PMC5568829; doi:10.15761/BGG.1000127)
Supplement: Table S2 [file NIHMS888767-supplement-Table_S2.pdf]

Table S2

pre-rehab vs. control

Gene	Affymetrix accession #	Gene description
GM-CSF Signaling		
AKT3	242876_at	v-akt murine thymoma viral oncogene homolog 3 (protein kinase B, gamma)
PIK3CD	203879_at	phosphoinositide-3-kinase, catalytic, delta polypeptide
ETS1	224833_at	v-ets erythroblastosis virus E26 oncogene homolog 1 (avian)
STAT1	AFFX-HUMISGF3A/M97935_3_at	signal transducer and activator of transcription 1, 91kDa
LYN	202626_s_at	v-yes-1 Yamaguchi sarcoma viral related oncogene homolog
SOS1	212780_at	son of sevenless homolog 1 (Drosophila)
KRAS	204009_s_at	v-Ki-ras2 Kirsten rat sarcoma viral oncogene homolog
GNB2L1	200651_at	guanine nucleotide binding protein (G protein), beta polypeptide 2-like 1
MAP2K2	213490_s_at	mitogen-activated protein kinase kinase 2
AKT2	225471_s_at	v-akt murine thymoma viral oncogene homolog 2
CAMK2G	212757_s_at	calcium/calmodulin-dependent protein kinase II gamma
CAMK2B	211483_x_at	calcium/calmodulin-dependent protein kinase II beta
MAPK1	208351_s_at	mitogen-activated protein kinase 1
PIK3R2	207105_s_at	phosphoinositide-3-kinase, regulatory subunit 2 (beta)
ATM	1553387_at	ataxia telangiectasia mutated
Protein Ubiquitination Pathway		
USP6	1555063_at	ubiquitin specific peptidase 6 (Tre-2 oncogene)
USP53	237465_at	ubiquitin specific peptidase 53
DNAJB8	237284_at	DnaJ (Hsp40) homolog, subfamily B, member 8
HSPA13	202557_at	heat shock protein 70kDa family, member 13
XIAP	206536_s_at	X-linked inhibitor of apoptosis
DNAJC21	230893_at	DnaJ (Hsp40) homolog, subfamily C, member 21
ANAPC10	241959_at	anaphase promoting complex subunit 10
DNAJB14	222850_s_at	DnaJ (Hsp40) homolog, subfamily B, member 14
SUGT1	224309_s_at	SGT1, suppressor of G2 allele of SKP1 (S. cerevisiae)
USP16	228822_s_at	ubiquitin specific peptidase 16
USP1	202412_s_at	ubiquitin specific peptidase 1
UBE2V2	209096_at	ubiquitin-conjugating enzyme E2 variant 2
UBE2N	212751_at	ubiquitin-conjugating enzyme E2N (UBC13 homolog, yeast)
USP9X	201099_at	ubiquitin specific peptidase 9, X-linked
PSMD6	202753_at	proteasome (prosome, macropain) 26S subunit, non-ATPase, 6
BAP1	1555735_a_at	BRCA1 associated protein-1 (ubiquitin carboxy-terminal hydrolase)
PSME1	200814_at	proteasome (prosome, macropain) activator subunit 1 (PA28 alpha)
USP48	220079_s_at	ubiquitin specific peptidase 48
PSMA2	201316_at	proteasome (prosome, macropain) subunit, alpha type, 2
UBE2B	239163_at	ubiquitin-conjugating enzyme E2B (RAD6 homolog)
PSMB5	208799_at	proteasome (prosome, macropain) subunit, beta type, 5
PSMD3	201388_at	proteasome (prosome, macropain) 26S subunit, non-ATPase, 3
USP21	218367_x_at	ubiquitin specific peptidase 21
TCEB2	200085_s_at	transcription elongation factor B (SIII), polypeptide 2 (18kDa, elongin B)
UBE2R2	223014_at	ubiquitin-conjugating enzyme E2R 2

DNAJC18	238115_at	DnaJ (Hsp40) homolog, subfamily C, member 18
STUB1	217934_x_at	STIP1 homology and U-box containing protein 1, E3 ubiquitin protein ligase
HSPA14	219212_at	heat shock 70kDa protein 14
DNAJC24	213853_at	DnaJ (Hsp40) homolog, subfamily C, member 24
DNAJC7	202416_at	DnaJ (Hsp40) homolog, subfamily C, member 7
USP19	214674_at	ubiquitin specific peptidase 19
UBE2D4	221949_at	ubiquitin-conjugating enzyme E2D 4 (putative)
USP27X	217605_at	ubiquitin specific peptidase 27, X-linked
PARK2	207058_s_at	parkinson protein 2, E3 ubiquitin protein ligase (parkin)
PSMB2	201404_x_at	proteasome (prosome, macropain) subunit, beta type, 2
NRAS	202647_s_at	neuroblastoma RAS viral (v-ras) oncogene homolog
CAMK2B	213276_at	calcium/calmodulin-dependent protein kinase II beta
PPP3CB	202432_at	protein phosphatase 3, catalytic subunit, beta isozyme
MAPK3	212046_x_at	mitogen-activated protein kinase 3
MAP2K2	202424_at	mitogen-activated protein kinase kinase 2

Glioma Signaling

AKT3	242876_at	v-akt murine thymoma viral oncogene homolog 3 (protein kinase B, gamma)
PIK3CD	203879_at	phosphoinositide-3-kinase, catalytic, delta polypeptide
PRKCI	213518_at	protein kinase C, iota
IGF1R	203628_at	insulin-like growth factor 1 receptor
SOS1	212780_at	son of sevenless homolog 1 (Drosophila)
KRAS	204009_s_at	v-Ki-ras2 Kirsten rat sarcoma viral oncogene homolog
CALM1 (includes others)	200653_s_at	calmodulin 1 (phosphorylase kinase, delta)
PDGFRA	203131_at	platelet-derived growth factor receptor, alpha polypeptide
PDGFB	216061_x_at	platelet-derived growth factor beta polypeptide (simian sarcoma viral (v-sis) oncogene homolog)
MAP2K2	213490_s_at	mitogen-activated protein kinase kinase 2
AKT2	225471_s_at	v-akt murine thymoma viral oncogene homolog 2
CAMK2G	212757_s_at	calcium/calmodulin-dependent protein kinase II gamma
CAMK2B	211483_x_at	calcium/calmodulin-dependent protein kinase II beta
MAPK1	208351_s_at	mitogen-activated protein kinase 1
CAMK1	1558556_at	calcium/calmodulin-dependent protein kinase I
CDKN2C	211792_s_at	cyclin-dependent kinase inhibitor 2C (p18, inhibits CDK4)
PIK3R2	207105_s_at	phosphoinositide-3-kinase, regulatory subunit 2 (beta)
ATM	1553387_at	ataxia telangiectasia mutated

FAK Signaling

AKT3	242876_at	v-akt murine thymoma viral oncogene homolog 3 (protein kinase B, gamma)
PIK3CD	203879_at	phosphoinositide-3-kinase, catalytic, delta polypeptide
PAK3	214607_at	p21 protein (Cdc42/Rac)-activated kinase 3
PTK2	241453_at	PTK2 protein tyrosine kinase 2
FYN	212486_s_at	FYN oncogene related to SRC, FGR, YES
SOS1	212780_at	son of sevenless homolog 1 (Drosophila)
KRAS	204009_s_at	v-Ki-ras2 Kirsten rat sarcoma viral oncogene homolog
WAS	38964_r_at	Wiskott-Aldrich syndrome (eczema-thrombocytopenia)
MAP2K2	213490_s_at	mitogen-activated protein kinase kinase 2
AKT2	225471_s_at	v-akt murine thymoma viral oncogene homolog 2

ACTA1	203872_at	actin, alpha 1, skeletal muscle
CAPN1	200752_s_at	calpain 1, (mu/I) large subunit
MAPK1	208351_s_at	mitogen-activated protein kinase 1
PAK2	208876_s_at	p21 protein (Cdc42/Rac)-activated kinase 2
PIK3R2	207105_s_at	phosphoinositide-3-kinase, regulatory subunit 2 (beta)
CAPN8	229030_at	calpain 8
ATM	1553387_at	ataxia telangiectasia mutated

CXCR4 Signaling

AKT3	242876_at	v-akt murine thymoma viral oncogene homolog 3 (protein kinase B, gamma)
RHOJ	243481_at	ras homolog gene family, member J
PIK3CD	203879_at	phosphoinositide-3-kinase, catalytic, delta polypeptide
ROCK2	211504_x_at	Rho-associated, coiled-coil containing protein kinase 2
GNAQ	224862_at	guanine nucleotide binding protein (G protein), q polypeptide
PAK3	214607_at	p21 protein (Cdc42/Rac)-activated kinase 3
PRKCI	213518_at	protein kinase C, iota
PTK2	241453_at	PTK2 protein tyrosine kinase 2
LYN	202626_s_at	v-yes-1 Yamaguchi sarcoma viral related oncogene homolog
KRAS	204009_s_at	v-Ki-ras2 Kirsten rat sarcoma viral oncogene homolog
GNB2L1	200651_at	guanine nucleotide binding protein (G protein), beta polypeptide 2-like 1
MYL2	209742_s_at	myosin, light chain 2, regulatory, cardiac, slow
MAP2K2	213490_s_at	mitogen-activated protein kinase kinase 2
GNAS	200780_x_at	GNAS complex locus
AKT2	225471_s_at	v-akt murine thymoma viral oncogene homolog 2
GNA11	204248_at	guanine nucleotide binding protein (G protein), alpha 11 (Gq class)
MYL12B	221474_at	myosin, light chain 12B, regulatory
RHOQ	212119_at	ras homolog gene family, member Q
MYL4	210395_x_at	myosin, light chain 4, alkali; atrial, embryonic
MAPK1	208351_s_at	mitogen-activated protein kinase 1
PAK2	208876_s_at	p21 protein (Cdc42/Rac)-activated kinase 2
MYL6B	204173_at	myosin, light chain 6B, alkali, smooth muscle and non-muscle
PIK3R2	207105_s_at	phosphoinositide-3-kinase, regulatory subunit 2 (beta)
ATM	1553387_at	ataxia telangiectasia mutated

3 wks rehab vs. control

Gene	Affymetrix accession #	Gene description
	Mitochondrial Dysfunction	
CASP3	202763_at	caspase 3, apoptosis-related cysteine peptidase
UCP2	208998_at	uncoupling protein 2 (mitochondrial, proton carrier)
PSEN1	203460_s_at	presenilin 1
COX15	221550_at	COX15 homolog, cytochrome c oxidase assembly protein (yeast)
APH1A	218389_s_at	anterior pharynx defective 1 homolog A (C. elegans)
ATP5C1	205711_x_at	ATP synthase, H+ transporting, mitochondrial F1 complex, gamma polypeptide 1
MT-COI	1553538_s_at	cytochrome c oxidase subunit I, Mitochondrion [Homo sapiens]
MT-CO2	1553570_x_at	mitochondrially encoded cytochrome c oxidase II
COX7B	202110_at	cytochrome c oxidase subunit VIIb
NDUFB9	222992_s_at	NADH dehydrogenase (ubiquinone) 1 beta subcomplex, 9, 22kDa
COX7C	201134_x_at	cytochrome c oxidase subunit VIIc
NDUFS4	209303_at	NADH dehydrogenase (ubiquinone) Fe-S protein 4, 18kDa (NADH-coenzyme Q reductase)
SDHB	214166_at	succinate dehydrogenase complex, subunit B, iron sulfur (Ip)
NDUFB8	201227_s_at	NADH dehydrogenase (ubiquinone) 1 beta subcomplex, 8, 19kDa
NDUFA2	209224_s_at	NADH dehydrogenase (ubiquinone) 1 alpha subcomplex, 2, 8kDa
NDUFS6	203606_at	NADH dehydrogenase (ubiquinone) Fe-S protein 6, 13kDa (NADH-coenzyme Q reductase)
NDUFB2	218200_s_at	NADH dehydrogenase (ubiquinone) 1 beta subcomplex, 2, 8kDa
NDUFB4	218226_s_at	NADH dehydrogenase (ubiquinone) 1 beta subcomplex, 4, 15kDa
NDUFA13	220864_s_at	NADH dehydrogenase (ubiquinone) 1 alpha subcomplex, 13
NDUFA6	202000_at	NADH dehydrogenase (ubiquinone) 1 alpha subcomplex, 6, 14kDa
NDUFB6	203613_s_at	NADH dehydrogenase (ubiquinone) 1 beta subcomplex, 6, 17kDa
TXN2	209077_at	thioredoxin 2
UQCRC1	201903_at	ubiquinol-cytochrome c reductase core protein I
NDUFA3	218563_at	NADH dehydrogenase (ubiquinone) 1 alpha subcomplex, 3, 9kDa
NDUFA11	228690_s_at	NADH dehydrogenase (ubiquinone) 1 alpha subcomplex, 11, 14.7kDa
NDUFA12	223244_s_at	NADH dehydrogenase (ubiquinone) 1 alpha subcomplex, 12
COX5A	203663_s_at	cytochrome c oxidase subunit Va
COX5B	202343_x_at	cytochrome c oxidase subunit Vb
NDUFB1	206790_s_at	NADH dehydrogenase (ubiquinone) 1 beta subcomplex, 1, 7kDa
COX8A	201119_s_at	cytochrome c oxidase subunit VIIIA (ubiquitous)
HTRA2	203089_s_at	HtrA serine peptidase 2
COX4I1	202698_x_at	cytochrome c oxidase subunit IV isoform 1
NDUFA8	218160_at	NADH dehydrogenase (ubiquinone) 1 alpha subcomplex, 8, 19kDa
CYCS	229415_at	cytochrome c, somatic
COX7A1	204570_at	cytochrome c oxidase subunit VIIa polypeptide 1 (muscle)
NDUFB11	218320_s_at	NADH dehydrogenase (ubiquinone) 1 beta subcomplex, 11, 17.3kDa
COX6B1	201441_at	cytochrome c oxidase subunit VIb polypeptide 1 (ubiquitous)
UQCRC2	241755_at	ubiquinol-cytochrome c reductase core protein II
NDUFS7	211752_s_at	NADH dehydrogenase (ubiquinone) Fe-S protein 7, 20kDa (NADH-coenzyme Q reductase)
SDHC	216591_s_at	succinate dehydrogenase complex, subunit C, integral membrane protein, 15kDa
NDUFB10	223112_s_at	NADH dehydrogenase (ubiquinone) 1 beta subcomplex, 10, 22kDa
COX6A2	206353_at	cytochrome c oxidase subunit VIa polypeptide 2
NDUFS1	235321_at	NADH dehydrogenase (ubiquinone) Fe-S protein 1, 75kDa (NADH-coenzyme Q reductase)
NDUFS8	203190_at	NADH dehydrogenase (ubiquinone) Fe-S protein 8, 23kDa (NADH-coenzyme Q reductase)

PARK2	207058_s_at	parkinson protein 2, E3 ubiquitin protein ligase (parkin)
	Oxidative Phosphorylation	
COX15	221550_at	COX15 homolog, cytochrome c oxidase assembly protein (yeast)
ATP6V1C1	226463_at	ATPase, H+ transporting, lysosomal 42kDa, V1 subunit C1
ATP5C1	205711_x_at	ATP synthase, H+ transporting, mitochondrial F1 complex, gamma polypeptide 1
MT-COI	1553538_s_at	cytochrome c oxidase subunit I, Mitochondrion [Homo sapiens]
MT-CO2	1553570_x_at	mitochondrially encoded cytochrome c oxidase II
COX7B	202110_at	cytochrome c oxidase subunit VIIb
NDUFB9	222992_s_at	NADH dehydrogenase (ubiquinone) 1 beta subcomplex, 9, 22kDa
COX7C	201134_x_at	cytochrome c oxidase subunit VIIc
NDUFS4	209303_at	NADH dehydrogenase (ubiquinone) Fe-S protein 4, 18kDa (NADH-coenzyme Q reductase)
SDHB	214166_at	succinate dehydrogenase complex, subunit B, iron sulfur (Ip)
NDUFC2	218101_s_at	NADH dehydrogenase (ubiquinone) 1, subcomplex unknown, 2, 14.5kDa
NDUFB8	201227_s_at	NADH dehydrogenase (ubiquinone) 1 beta subcomplex, 8, 19kDa
ATP5G2	208764_s_at	ATP synthase, H+ transporting, mitochondrial Fo complex, subunit C2 (subunit 9)
NDUFA2	209224_s_at	NADH dehydrogenase (ubiquinone) 1 alpha subcomplex, 2, 8kDa
NDUFS6	203606_at	NADH dehydrogenase (ubiquinone) Fe-S protein 6, 13kDa (NADH-coenzyme Q reductase)
NDUFB2	218200_s_at	NADH dehydrogenase (ubiquinone) 1 beta subcomplex, 2, 8kDa
NDUFB4	218226_s_at	NADH dehydrogenase (ubiquinone) 1 beta subcomplex, 4, 15kDa
UQCR10	218190_s_at	ubiquinol-cytochrome c reductase, complex III subunit X
NDUFA13	220864_s_at	NADH dehydrogenase (ubiquinone) 1 alpha subcomplex, 13
NDUFA6	202000_at	NADH dehydrogenase (ubiquinone) 1 alpha subcomplex, 6, 14kDa
NDUFB6	203613_s_at	NADH dehydrogenase (ubiquinone) 1 beta subcomplex, 6, 17kDa
UQCRC1	201903_at	ubiquinol-cytochrome c reductase core protein I
NDUFA3	218563_at	NADH dehydrogenase (ubiquinone) 1 alpha subcomplex, 3, 9kDa
NDUFA11	228690_s_at	NADH dehydrogenase (ubiquinone) 1 alpha subcomplex, 11, 14.7kDa
NDUFA12	223244_s_at	NADH dehydrogenase (ubiquinone) 1 alpha subcomplex, 12
COX5A	203663_s_at	cytochrome c oxidase subunit Va
COX5B	202343_x_at	cytochrome c oxidase subunit Vb
ATP5L	207573_x_at	ATP synthase, H+ transporting, mitochondrial Fo complex, subunit G
NDUFB1	206790_s_at	NADH dehydrogenase (ubiquinone) 1 beta subcomplex, 1, 7kDa
COX8A	201119_s_at	cytochrome c oxidase subunit VIIIA (ubiquitous)
COX4I1	202698_x_at	cytochrome c oxidase subunit IV isoform 1
ATP5F1	243501_at	ATP synthase, H+ transporting, mitochondrial Fo complex, subunit B1
NDUFA8	218160_at	NADH dehydrogenase (ubiquinone) 1 alpha subcomplex, 8, 19kDa
COX7A1	204570_at	cytochrome c oxidase subunit VIIa polypeptide 1 (muscle)
NDUFB11	218320_s_at	NADH dehydrogenase (ubiquinone) 1 beta subcomplex, 11, 17.3kDa
COX6B1	201441_at	cytochrome c oxidase subunit VIb polypeptide 1 (ubiquitous)
ATP5H	210149_s_at	ATP synthase, H+ transporting, mitochondrial Fo complex, subunit d
UQCRC2	241755_at	ubiquinol-cytochrome c reductase core protein II
NDUFS7	211752_s_at	NADH dehydrogenase (ubiquinone) Fe-S protein 7, 20kDa (NADH-coenzyme Q reductase)
SDHC	216591_s_at	succinate dehydrogenase complex, subunit C, integral membrane protein, 15kDa
NDUFB10	223112_s_at	NADH dehydrogenase (ubiquinone) 1 beta subcomplex, 10, 22kDa
COX6A2	206353_at	cytochrome c oxidase subunit VIa polypeptide 2
NDUFS1	235321_at	NADH dehydrogenase (ubiquinone) Fe-S protein 1, 75kDa (NADH-coenzyme Q reductase)
NDUFS8	203190_at	NADH dehydrogenase (ubiquinone) Fe-S protein 8, 23kDa (NADH-coenzyme Q reductase)

	Actin Cytoskeleton Signaling	
ITGA4	213416_at	integrin, alpha 4 (antigen CD49D, alpha 4 subunit of VLA-4 receptor)
MYL5	205145_s_at	myosin, light chain 5, regulatory
PIK3C2A	1569021_at	phosphoinositide-3-kinase, class 2, alpha polypeptide
F2R	203989_x_at	coagulation factor II (thrombin) receptor
IQGAP2	203474_at	IQ motif containing GTPase activating protein 2
RAC2	213603_s_at	ras-related C3 botulinum toxin substrate 2 (rho family, small GTP binding protein Rac2)
PIK3CD	203879_at	phosphoinositide-3-kinase, catalytic, delta polypeptide
CD14	201743_at	CD14 molecule
FGF12	207501_s_at	fibroblast growth factor 12
VAV2	226063_at	vav 2 guanine nucleotide exchange factor
EZR	208621_s_at	ezrin
PDGFD	219304_s_at	platelet derived growth factor D
ACTN1	208636_at	actinin, alpha 1
CDC42	214230_at	cell division cycle 42 (GTP binding protein, 25kDa)
NCKAP1	207738_s_at	NCK-associated protein 1
LIMK2	202193_at	LIM domain kinase 2
ARPC1B	201954_at	actin related protein 2/3 complex, subunit 1B, 41kDa
GNG12	212294_at	guanine nucleotide binding protein (G protein), gamma 12
MSN	200600_at	moesin
IQGAP1	200791_s_at	IQ motif containing GTPase activating protein 1
ROCK2	211504_x_at	Rho-associated, coiled-coil containing protein kinase 2
PFN4	235627_at	profilin family, member 4
NRAS	224985_at	neuroblastoma RAS viral (v-ras) oncogene homolog
TMSB10/TMSB4X	217733_s_at	thymosin beta 10
ACTB	200801_x_at	actin, beta
ARPC2	207988_s_at	actin related protein 2/3 complex, subunit 2, 34kDa
PAK2	208877_at	p21 protein (Cdc42/Rac)-activated kinase 2
DIAPH2	205726_at	diaphanous homolog 2 (Drosophila)
ACTG1	224585_x_at	actin, gamma 1
WASL	224813_at	Wiskott-Aldrich syndrome-like
MAPK1	212271_at	mitogen-activated protein kinase 1
PPP1CB	201407_s_at	protein phosphatase 1, catalytic subunit, beta isozyme
WASF2	224562_at	WAS protein family, member 2
ACTR3	200996_at	ARP3 actin-related protein 3 homolog (yeast)
MYH10	213067_at	myosin, heavy chain 10, non-muscle
CYFIP1	208923_at	cytoplasmic FMR1 interacting protein 1
ACTR2	200728_at	ARP2 actin-related protein 2 homolog (yeast)
APC	203525_s_at	adenomatous polyposis coli
PPP1R12A	201602_s_at	protein phosphatase 1, regulatory (inhibitor) subunit 12A
PIK3CA	204369_at	phosphoinositide-3-kinase, catalytic, alpha polypeptide
ITGB1	211945_s_at	integrin, beta 1 (fibronectin receptor, beta polypeptide, antigen CD29 includes MDF2, MSK12)
C3orf10	224023_s_at	chromosome 3 open reading frame 10
TTN	208195_at	titin
MYL2	209742_s_at	myosin, light chain 2, regulatory, cardiac, slow
PIK3C2B	204484_at	phosphoinositide-3-kinase, class 2, beta polypeptide
CRKL	212180_at	v-crk sarcoma virus CT10 oncogene homolog (avian)-like
MYH7	216265_x_at	myosin, light chain 7, regulatory

MYL4	210395_x_at	myosin, light chain 4, alkali; atrial, embryonic
MAP2K2	213490_s_at	mitogen-activated protein kinase kinase 2
GNA12	221737_at	guanine nucleotide binding protein (G protein) alpha 12
ABI2	216113_at	abl-interactor 2
ACTN2	203864_s_at	actinin, alpha 2
MYLK3	238834_at	myosin light chain kinase 3
MYL6B	204173_at	myosin, light chain 6B, alkali, smooth muscle and non-muscle
MYH7B	215795_at	myosin, heavy chain 7B, cardiac muscle, beta
	Integrin Signaling	
ITGA4	213416_at	integrin, alpha 4 (antigen CD49D, alpha 4 subunit of VLA-4 receptor)
MYL5	205145_s_at	myosin, light chain 5, regulatory
CAPN6	202965_s_at	calpain 6
PIK3C2A	1569021_at	phosphoinositide-3-kinase, class 2, alpha polypeptide
ITGAL	213475_s_at	integrin, alpha L (antigen CD11A (p180), lymphocyte function-associated antigen 1; alpha polypeptide)
RAC2	213603_s_at	ras-related C3 botulinum toxin substrate 2 (rho family, small GTP binding protein Rac2)
PIK3CD	203879_at	phosphoinositide-3-kinase, catalytic, delta polypeptide
ITGB2	202803_s_at	integrin, beta 2 (complement component 3 receptor 3 and 4 subunit)
ITGAM	205786_s_at	integrin, alpha M (complement component 3 receptor 3 subunit)
WIPF1	202664_at	WAS/WASL interacting protein family, member 1
PTEN	211711_s_at	phosphatase and tensin homolog
RAP2B	213923_at	RAP2B, member of RAS oncogene family
ACTN1	208636_at	actinin, alpha 1
NEDD9	202150_s_at	neural precursor cell expressed, developmentally down-regulated 9
DIRAS3	215506_s_at	DIRAS family, GTP-binding RAS-like 3
CDC42	214230_at	cell division cycle 42 (GTP binding protein, 25kDa)
FYN	210105_s_at	FYN oncogene related to SRC, FGR, YES
ARPC1B	201954_at	actin related protein 2/3 complex, subunit 1B, 41kDa
RAP1B	200833_s_at	RAP1B, member of RAS oncogene family
ASAP1	224791_at	ArfGAP with SH3 domain, ankyrin repeat and PH domain 1
NRAS	224985_at	neuroblastoma RAS viral (v-ras) oncogene homolog
ACTB	200801_x_at	actin, beta
RHOJ	235489_at	ras homolog gene family, member J
ARPC2	207988_s_at	actin related protein 2/3 complex, subunit 2, 34kDa
CAV1	212097_at	caveolin 1, caveolae protein, 22kDa
PAK2	208877_at	p21 protein (Cdc42/Rac)-activated kinase 2
ARF3	200734_s_at	ADP-ribosylation factor 3
ACTG1	224585_x_at	actin, gamma 1
CAPN2	208683_at	calpain 2, (m/II) large subunit
RHOG	203175_at	ras homolog gene family, member G (rho G)
WASL	224813_at	Wiskott-Aldrich syndrome-like
MAPK1	212271_at	mitogen-activated protein kinase 1
PPP1CB	201407_s_at	protein phosphatase 1, catalytic subunit, beta isozyme
ACTR3	200996_at	ARP3 actin-related protein 3 homolog (yeast)
ITGA6	215177_s_at	integrin, alpha 6
ACTR2	200728_at	ARP2 actin-related protein 2 homolog (yeast)
PPP1R12A	201602_s_at	protein phosphatase 1, regulatory (inhibitor) subunit 12A
PIK3CA	204369_at	phosphoinositide-3-kinase, catalytic, alpha polypeptide

ITGB1	211945_s_at	integrin, beta 1 (fibronectin receptor, beta polypeptide, antigen CD29 includes MDF2, MSK12)
NCK1	211063_s_at	NCK adaptor protein 1
TTN	208195_at	titin
MYL2	209742_s_at	myosin, light chain 2, regulatory, cardiac, slow
PIK3C2B	204484_at	phosphoinositide-3-kinase, class 2, beta polypeptide
TSPAN4	209264_s_at	tetraspanin 4
CRKL	212180_at	v-crk sarcoma virus CT10 oncogene homolog (avian)-like
MAP2K2	213490_s_at	mitogen-activated protein kinase kinase 2
ACTN2	203864_s_at	actinin, alpha 2
MYLK3	238834_at	myosin light chain kinase 3
GSK3B	242336_at	glycogen synthase kinase 3 beta
	Ubiquinone Biosynthesis	
PRMT1	206445_s_at	protein arginine methyltransferase 1
NDUFB9	222992_s_at	NADH dehydrogenase (ubiquinone) 1 beta subcomplex, 9, 22kDa
NDUFS4	209303_at	NADH dehydrogenase (ubiquinone) Fe-S protein 4, 18kDa (NADH-coenzyme Q reductase)
NDUFC2	218101_s_at	NADH dehydrogenase (ubiquinone) 1, subcomplex unknown, 2, 14.5kDa
NDUFB8	201227_s_at	NADH dehydrogenase (ubiquinone) 1 beta subcomplex, 8, 19kDa
NDUFA2	209224_s_at	NADH dehydrogenase (ubiquinone) 1 alpha subcomplex, 2, 8kDa
NDUFS6	203606_at	NADH dehydrogenase (ubiquinone) Fe-S protein 6, 13kDa (NADH-coenzyme Q reductase)
NDUFB2	218200_s_at	NADH dehydrogenase (ubiquinone) 1 beta subcomplex, 2, 8kDa
NDUFB4	218226_s_at	NADH dehydrogenase (ubiquinone) 1 beta subcomplex, 4, 15kDa
NDUFA13	220864_s_at	NADH dehydrogenase (ubiquinone) 1 alpha subcomplex, 13
NDUFA6	202000_at	NADH dehydrogenase (ubiquinone) 1 alpha subcomplex, 6, 14kDa
NDUFB6	203613_s_at	NADH dehydrogenase (ubiquinone) 1 beta subcomplex, 6, 17kDa
NDUFA3	218563_at	NADH dehydrogenase (ubiquinone) 1 alpha subcomplex, 3, 9kDa
NDUFA11	228690_s_at	NADH dehydrogenase (ubiquinone) 1 alpha subcomplex, 11, 14.7kDa
NDUFA12	223244_s_at	NADH dehydrogenase (ubiquinone) 1 alpha subcomplex, 12
NDUFB1	206790_s_at	NADH dehydrogenase (ubiquinone) 1 beta subcomplex, 1, 7kDa
NDUFA8	218160_at	NADH dehydrogenase (ubiquinone) 1 alpha subcomplex, 8, 19kDa
NDUFB11	218320_s_at	NADH dehydrogenase (ubiquinone) 1 beta subcomplex, 11, 17.3kDa
NDUFS7	211752_s_at	NADH dehydrogenase (ubiquinone) Fe-S protein 7, 20kDa (NADH-coenzyme Q reductase)
NDUFB10	223112_s_at	NADH dehydrogenase (ubiquinone) 1 beta subcomplex, 10, 22kDa
NDUFS1	235321_at	NADH dehydrogenase (ubiquinone) Fe-S protein 1, 75kDa (NADH-coenzyme Q reductase)
NDUFS8	203190_at	NADH dehydrogenase (ubiquinone) Fe-S protein 8, 23kDa (NADH-coenzyme Q reductase)

6 wks rehab vs. control

Gene	Affymetrix accession #	Gene description
Mitochondrial Dysfunction		
COX11	214277_at	COX11 cytochrome c oxidase assembly homolog (yeast)
LRRK2	229584_at	leucine-rich repeat kinase 2
APP	200602_at	amyloid beta (A4) precursor protein
MAPK8	229664_at	mitogen-activated protein kinase 8
PINK1	209019_s_at	PTEN induced putative kinase 1
PSEN1	203460_s_at	presenilin 1
TXNRD2	211177_s_at	thioredoxin reductase 2
NDUFA12	223244_s_at	NADH dehydrogenase (ubiquinone) 1 alpha subcomplex, 12
NDUFB1	206790_s_at	NADH dehydrogenase (ubiquinone) 1 beta subcomplex, 1, 7kDa
NDUFA13	220864_s_at	NADH dehydrogenase (ubiquinone) 1 alpha subcomplex, 13
ATP5C1	205711_x_at	ATP synthase, H+ transporting, mitochondrial F1 complex, gamma polypeptide 1
NDUFB10	223112_s_at	NADH dehydrogenase (ubiquinone) 1 beta subcomplex, 10, 22kDa
FIS1	218034_at	fission 1 (mitochondrial outer membrane) homolog (S. cerevisiae
NDUFB8	214241_at	NADH dehydrogenase (ubiquinone) 1 beta subcomplex, 8, 19kDa
NDUFB4	218226_s_at	NADH dehydrogenase (ubiquinone) 1 beta subcomplex, 4, 15kDa
COX6A2	206353_at	cytochrome c oxidase subunit VIa polypeptide 2
COX8A	201119_s_at	cytochrome c oxidase subunit VIIIA (ubiquitous)
PDHA1	200980_s_at	pyruvate dehydrogenase (lipoamide) alpha 1
MAP2K4	203265_s_at	mitogen-activated protein kinase kinase 4
NDUFS8	203189_s_at	NADH dehydrogenase (ubiquinone) Fe-S protein 8, 23kDa (NADH-coenzyme Q reductase)
BACE1	224335_s_at	beta-site APP-cleaving enzyme 1
PARK2	207058_s_at	parkinson protein 2, E3 ubiquitin protein ligase (parkin)
OGDH	1554151_at	oxoglutarate (alpha-ketoglutarate) dehydrogenase (lipoamide)
Integrin Signaling		
ITGA4	213416_at	integrin, alpha 4 (antigen CD49D, alpha 4 subunit of VLA-4 receptor)
PIK3CD	203879_at	phosphoinositide-3-kinase, catalytic, delta polypeptide
WIPF1	202664_at	WAS/WASL interacting protein family, member 1
NRAS	202647_s_at	neuroblastoma RAS viral (v-ras) oncogene homolog
PTEN	211711_s_at	phosphatase and tensin homolog
PPP1CB	201407_s_at	protein phosphatase 1, catalytic subunit, beta isozyme
ACTR3	213102_at	ARP3 actin-related protein 3 homolog (yeast)
RAP1B	200833_s_at	RAP1B, member of RAS oncogene family
PAK2	1559052_s_at	p21 protein (Cdc42/Rac)-activated kinase 2
RAP2A	225585_at	RAP2A, member of RAS oncogene family
RALA	224880_at	v-ral simian leukemia viral oncogene homolog A (ras related)
MAPK8	229664_at	mitogen-activated protein kinase 8
ARHGEF7	202547_s_at	Rho guanine nucleotide exchange factor (GEF) 7
RAC1	1567458_s_at	ras-related C3 botulinum toxin substrate 1 (rho family, small GTP binding protein Rac1)
ARF4	201097_s_at	ADP-ribosylation factor 4
SOS1	212780_at	son of sevenless homolog 1 (Drosophila)
RHOQ	212119_at	ras homolog gene family, member Q
PIK3R4	212740_at	phosphoinositide-3-kinase, regulatory subunit 4
ACTN2	203864_s_at	actinin, alpha 2

MAP2K4	203265_s_at	mitogen-activated protein kinase kinase 4
RHOJ	235131_at	ras homolog gene family, member J
MAP2K2	213490_s_at	mitogen-activated protein kinase kinase 2
ACTN2	203863_at	actinin, alpha 2
CAPN1	200752_s_at	calpain 1, (mu/I) large subunit
CAPN3	211890_x_at	calpain 3, (p94)
ATM	1553387_at	ataxia telangiectasia mutated
GSK3B	242336_at	glycogen synthase kinase 3 beta
GSK3B	242336_at	glycogen synthase kinase 3 beta
PAK1	1565772_at	p21 protein (Cdc42/Rac)-activated kinase 1
ITGB3	204627_s_at	integrin, beta 3 (platelet glycoprotein IIIa, antigen CD61)

Ephrin Receptor Signaling

ITGA4	213416_at	integrin, alpha 4 (antigen CD49D, alpha 4 subunit of VLA-4 receptor)
ATF2	205446_s_at	activating transcription factor 2
WIPF1	202664_at	WAS/WASL interacting protein family, member 1
GNAL	206355_at	guanine nucleotide binding protein (G protein), alpha activating activity polypeptide, olfactory type
NRAS	202647_s_at	neuroblastoma RAS viral (v-ras) oncogene homolog
DOK1	216835_s_at	docking protein 1, 62kDa (downstream of tyrosine kinase 1)
CREB1	204313_s_at	cAMP responsive element binding protein 1
GNA13	227539_at	guanine nucleotide binding protein (G protein), alpha 13
CXCL12	209687_at	chemokine (C-X-C motif) ligand 12
ACTR3	213102_at	ARP3 actin-related protein 3 homolog (yeast)
PDGFC	218718_at	platelet derived growth factor C
RAP1B	200833_s_at	RAP1B, member of RAS oncogene family
PAK2	1559052_s_at	p21 protein (Cdc42/Rac)-activated kinase 2
MAP4K4	222547_at	mitogen-activated protein kinase kinase kinase kinase 4
GNG10	201921_at	DNAJC25-GNG10 readthrough
SDCBP	200958_s_at	syndecan binding protein (syntenin)
RASA1	210621_s_at	RAS p21 protein activator (GTPase activating protein) 1
RAC1	1567458_s_at	ras-related C3 botulinum toxin substrate 1 (rho family, small GTP binding protein Rac1)
SOS1	212780_at	son of sevenless homolog 1 (Drosophila)
GRINA	212090_at	glutamate receptor, ionotropic, N-methyl D-aspartate-associated protein 1 (glutamate binding)
GNAS	200981_x_at	GNAS complex locus
MAP2K2	213490_s_at	mitogen-activated protein kinase kinase 2
EFNB2	202668_at	ephrin-B2
PAK1	1565772_at	p21 protein (Cdc42/Rac)-activated kinase 1

HMGB1 Signaling

PIK3CD	203879_at	phosphoinositide-3-kinase, catalytic, delta polypeptide
IL1R1	202948_at	interleukin 1 receptor, type I
NRAS	202647_s_at	neuroblastoma RAS viral (v-ras) oncogene homolog
KAT2B	239585_at	K(lysine) acetyltransferase 2B
MAPK8	229664_at	mitogen-activated protein kinase 8
RAC1	1567458_s_at	ras-related C3 botulinum toxin substrate 1 (rho family, small GTP binding protein Rac1)
MYST2	200049_at	MYST histone acetyltransferase 2
HMGB1	200679_x_at	high-mobility group box 1
RHOQ	212119_at	ras homolog gene family, member Q

PIK3R4	212740_at	phosphoinositide-3-kinase, regulatory subunit 4
MAP2K4	203265_s_at	mitogen-activated protein kinase kinase 4
RHOJ	235131_at	ras homolog gene family, member J
MAP2K2	213490_s_at	mitogen-activated protein kinase kinase 2
ATM	1553387_at	ataxia telangiectasia mutated
ELK1	203617_x_at	ELK1, member of ETS oncogene family
MAP2K7	216206_x_at	mitogen-activated protein kinase kinase 7

Rac Signaling

ITGA4	213416_at	integrin, alpha 4 (antigen CD49D, alpha 4 subunit of VLA-4 receptor)
PIK3CD	203879_at	phosphoinositide-3-kinase, catalytic, delta polypeptide
WASF1	204165_at	WAS protein family, member 1
NRAS	202647_s_at	neuroblastoma RAS viral (v-ras) oncogene homolog
ACTR3	213102_at	ARP3 actin-related protein 3 homolog (yeast)
PAK2	1559052_s_at	p21 protein (Cdc42/Rac)-activated kinase 2
MAPK8	229664_at	mitogen-activated protein kinase 8
RAC1	1567458_s_at	ras-related C3 botulinum toxin substrate 1 (rho family, small GTP binding protein Rac1)
ANK1	207087_x_at	ankyrin 1, erythrocytic
PIK3R4	212740_at	phosphoinositide-3-kinase, regulatory subunit 4
MAP2K4	203265_s_at	mitogen-activated protein kinase kinase 4
MAP2K2	213490_s_at	mitogen-activated protein kinase kinase 2
ATM	1553387_at	ataxia telangiectasia mutated
ELK1	203617_x_at	ELK1, member of ETS oncogene family
MAP2K7	216206_x_at	mitogen-activated protein kinase kinase 7
CD44	216062_at	CD44 molecule (Indian blood group)
PAK1	1565772_at	p21 protein (Cdc42/Rac)-activated kinase 1
