# Supplementary material for: Molecular signatures of differential responses to exercise trainings during rehabilitation
Source: Biomed Genet Genom. Author manuscript; Available in PMC 2017 Aug 23. (PMC5568829; doi:10.15761/BGG.1000127)
Supplement: Table S3 [file NIHMS888767-supplement-Table_S3.pdf]

Table S3 A cluster of genes identified by hierarchical clustering to be differentially regulated in the high responders

**Affymetrix accession #**	**Gene description**	**Fold change Immobilization**		**Fold change Rehabilitation (3 weeks)**		**Fold change Rehabilitation (6 weeks)**	
		**High Responders**	**Low Responders**	**High Responders**	**Low Responders**	**High Responders**	**Low Responders**
221518_s_at	601659921R1 NIH_MGC_71 Homo sapiens cDNA clone IMAGE:3905741 3', mRNA sequence.	NA	NA	-1.1	NA	NA	NA
225783_at	602083944F1 NIH_MGC_83 Homo sapiens cDNA clone IMAGE:4248227 5', mRNA sequence.	NA	NA	-1.2	NA	NA	NA

241959_at	anaphase-promoting complex subunit 10	NA	1.3	NA	NA	NA	NA
206536_s_at	baculoviral IAP repeat-containing 4	NA	1.3	NA	NA	NA	NA
202387_at	BCL2-associated athanogene	-1.3	NA	-1.2	NA	-1.3	NA
220372_at	chromosome 21 open reading frame 55	-1.4	NA	-1.4	NA	-1.3	NA
209283_at	crystallin, alpha B	-1.2	NA	-1.2	NA	NA	NA
203079_s_at	cullin 2	-1.1	NA	-1.2	NA	-1.1	NA
202500_at	DnaJ (Hsp40) homolog, subfamily B, member 2	NA	NA	-1.2	NA	NA	NA
203811_s_at	DnaJ (Hsp40) homolog, subfamily B, member 4	NA	NA	-1.4	NA	NA	NA
209015_s_at	DnaJ (Hsp40) homolog, subfamily B, member 6	NA	NA	-1.4	NA	-1.2	NA

237284_at	DnaJ (Hsp40) homolog, subfamily B, member 8	NA	1.5	NA	NA	NA	NA
202842_s_at	DnaJ (Hsp40) homolog, subfamily B, member 9	NA	NA	-1.2	NA	NA	NA
218409_s_at	DnaJ (Hsp40) homolog, subfamily C, member 1	-1.3	NA	NA	NA	NA	NA
212490_at	DnaJ (Hsp40) homolog, subfamily C, member 8	NA	NA	-1.2	NA	NA	NA
1554451_s_at	DnaJ protein	-1.5	NA	-1.8	NA	NA	NA
206976_s_at	heat shock 105kDa/110kDa protein 1	NA	NA	-1.3	NA	NA	NA
205133_s_at	heat shock 10kDa protein 1 (chaperonin 10)	NA	NA	-1.3	NA	NA	NA
208687_x_at	heat shock 70kDa protein 8	NA	NA	-1.4	NA	NA	NA

200690_at	heat shock 70kDa protein 9B (mortalin-2)	NA	NA	-1.4	NA	-1.2	NA
210211_s_at	heat shock 90kDa protein 1, alpha	NA	NA	-1.2	NA	NA	NA
200064_at	heat shock 90kDa protein 1, beta	NA	NA	-1.2	-1.1	-1.1	NA
224309_s_at	Homo sapiens HDCMD34P mRNA, complete cds.	NA	1.2	-1.1	NA	-1.2	NA
200806_s_at	Homo sapiens transcribed sequence with strong similarity to protein pir:A32800 (H.sapiens) A32800 chaperonin GroEL precursor - human	NA	NA	-1.3	NA	NA	NA
219861_at	hypothetical protein FLJ10634	-1.4	NA	NA	NA	NA	NA
223721_s_at	J domain containing protein 1	-1.4	NA	NA	NA	NA	NA
237465_at	KIAA1350 protein	NA	1.9	NA	NA	NA	NA

204219_s_at	proteasome (prosome, macropain) 26S subunit, ATPase, 1	NA	NA	-1.2	NA	NA	NA
201067_at	proteasome (prosome, macropain) 26S subunit, ATPase, 2	NA	NA	-1.2	-1.1	-1.1	NA
208777_s_at	proteasome (prosome, macropain) 26S subunit, non-ATPase, 11	NA	NA	-1.3	NA	NA	NA
202352_s_at	proteasome (prosome, macropain) 26S subunit, non-ATPase, 12	NA	NA	-1.2	NA	NA	NA
201532_at	proteasome (prosome, macropain) subunit, alpha type, 3	NA	NA	-1.2	NA	NA	NA
201274_at	proteasome (prosome, macropain) subunit, alpha type, 5	-1.2	NA	-1.2	NA	NA	NA
200876_s_at	proteasome (prosome, macropain) subunit, beta type, 1	-1.1	NA	-1.1	NA	-1.1	NA

211609_x_at	Subunit of the 26S protease that binds and presumably selects ubiquitin-conjugates for destruction; Subunit 5a of the 26S protease regulatory complex; Human 26S protease subunit S5a mRNA, complete cds.	NA	NA	-1.2	NA	-1.2	NA
203235_at	thimet oligopeptidase 1	-1.4	NA	-1.3	NA	NA	NA
227788_at	ubiquitin specific protease 13 (isopeptidase T-3)	NA	NA	-1.2	NA	NA	NA
228822_s_at	ubiquitin specific protease 16	NA	1.2	NA	NA	NA	1.2
212381_at	ubiquitin specific protease 24	NA	NA	-1.3	NA	NA	NA
209096_at	ubiquitin-conjugating enzyme E2 variant 2	NA	1.2	NA	1.1	NA	1.1

201898_s_at	ubiquitin-conjugating enzyme E2A (RAD6 homolog)	-1.2	NA	NA	NA	-1.1	NA
200668_s_at	ubiquitin-conjugating enzyme E2D 3 (UBC4/5 homolog, yeast)	-1.1	NA	-1.2	NA	NA	NA
200676_s_at	ubiquitin-conjugating enzyme E2L 3	NA	NA	-1.3	NA	NA	NA
212751_at	ubiquitin-conjugating enzyme E2N (UBC13 homolog, yeast)	-1.1	1.1	NA	NA	NA	NA
